# Genome-Wide Association Study with Three Control Cohorts of Japanese Patients with Esotropia and Exotropia of Comitant Strabismus and Idiopathic Superior Oblique Muscle Palsy

**DOI:** 10.3390/ijms25136986

**Published:** 2024-06-26

**Authors:** Toshihiko Matsuo, Ichiro Hamasaki, Yoichiro Kamatani, Takahisa Kawaguchi, Izumi Yamaguchi, Fumihiko Matsuda, Akira Saito, Kazuyuki Nakazono, Shigeo Kamitsuji

**Affiliations:** 1 Graduate School of Interdisciplinary Science and Engineering in Health Systems, Okayama University, Okayama City 700-8558, Japan; 2 Department of Ophthalmology, Okayama University Hospital, Okayama City 700-8558, Japan; 3 Department of Computational Biology and Medical Sciences, Graduate School of Frontier Sciences, The University of Tokyo, Tokyo 108-8639, Japan; kamatani.yoichiro@edu.k.u-tokyo.ac.jp; 4 Center for Genomic Medicine, Graduate School of Medicine, Kyoto University, Kyoto 606-8507, Japanfumi@genome.med.kyoto-u.ac.jp (F.M.); 5 StaGen Co., Ltd., Tokyo 111-0051, Japankamitsuji@stagen.co.jp (S.K.)

**Keywords:** esotropia, exotropia, superior oblique muscle palsy, genome-wide association study, comitant strabismus, non-comitant strabismus, Japanese population, BioBank Japan, Nagahama Cohort, Asian array

## Abstract

Esotropia and exotropia in the entity of comitant strabismus are multifactorial diseases with both genetic and environmental backgrounds. Idiopathic superior oblique muscle palsy, as the predominant entity of non-comitant (paralytic) strabismus, also has a genetic background, as evidenced by varying degrees of muscle hypoplasia. A genome-wide association study (GWAS) was conducted of 711 Japanese patients with esotropia (n= 253), exotropia (n = 356), and idiopathic superior oblique muscle palsy (n = 102). The genotypes of single nucleotide polymorphisms (SNPs) were determined by Infinium Asian Screening Array. Three control cohorts from the Japanese population were used: two cohorts from BioBank Japan (BBJ) and the Nagahama Cohort. BBJ (180K) was genotyped by a different array, Illumina Infinium OmniExpressExome or HumanOmniExpress, while BBJ (ASA) and the Nagahama Cohort were genotyped by the same Asian array. After quality control of SNPs and individuals, common SNPs between the case cohort and the control cohort were chosen in the condition of genotyping by different arrays, while all SNPs genotyped by the same array were used for SNP imputation. The SNPs imputed with R-square values ≥ 0.3 were used to compare the case cohort of each entity or the combined entity with the control cohort. In comparison with BBJ (180K), the esotropia group and the exotropia group showed *CDCA7* and *HLA-F*, respectively, as candidate genes at a significant level of *p* < 5 × 10^−8^, while the idiopathic superior oblique muscle palsy group showed *DAB1* as a candidate gene which is involved in neuronal migration. *DAB1* was also detected as a candidate in comparison with BBJ (ASA) and the Nagahama Cohort at a weak level of significance of *p* < 1 × 10^−6^. In comparison with BBJ (180K), *RARB* (retinoic acid receptor-β) was detected as a candidate at a significant level of *p* < 5 × 10^−8^ in the combined group of esotropia, exotropia, and idiopathic superior oblique muscle palsy. In conclusion, a series of GWASs with three different control cohorts would be an effective method with which to search for candidate genes for multifactorial diseases such as strabismus.

## 1. Introduction

Both eyes are located at the front of the head in humans and some animals, and this results in the overlapped visual fields of two eyes, and thus generates binocular vision, which consists of three levels: simultaneous perception, binocular fusion, and stereopsis. Strabismus is a disease of binocular misalignment in which both eyes do not align with the same visual target, leading to abnormal binocular vision, such as monocular suppression, double vision, and a lack of stereopsis. Two entities of strabismus are comitant strabismus and non-comitant (paralytic) strabismus. Comitant strabismus shows the same degree of eye deviation in all directions of the gaze, while non-comitant strabismus shows different degrees of deviation according to the different directions of the gaze. Major types of comitant strabismus are esotropia and exotropia, which present as inward and outward deviations, respectively, of one eye relative to the other eye along the horizontal axis [1]. Major types of non-comitant strabismus involve the paralysis of ocular motor nerves, including the abducens, oculomotor, and trochlear nerves, which innervate the extraocular muscles [2].

Esotropia and exotropia, as forms of comitant strabismus, are fundamentally early-onset in life [3,4,5,6,7] and are multifactorial diseases which have both genetic and environmental background factors [8,9,10]. The prevalence of comitant strabismus is estimated at roughly 1–2% of the population [4,5]. The genetic background for comitant strabismus is supported by the presence of family history [11,12] and the concordance of phenotypes in monozygotic twins [13,14], while an environmental background is suggested by perinatal hypoxia [15,16]. Idiopathic superior oblique muscle palsy, as a form of non-comitant strabismus, manifests in the earlier years of life as congenital palsy or later in life as the decompensation of binocular vision in the process of aging [2,17,18]. The prevalence of idiopathic superior oblique muscle palsy remains to be elucidated and is empirically considered to be lower by an order of magnitude than comitant strabismus. A genetic background is highly suspected in idiopathic superior oblique muscle palsy since varying degrees of hypoplasia of the superior oblique muscle have been disclosed by orbital magnetic resonance imaging [19,20,21,22,23,24].

Linkage analyses by chromosomal mapping to find susceptibility loci for esotropia and exotropia of comitant strabismus have been conducted in families from different populations [25,26,27,28,29,30,31,32]. Linkage analysis has also been performed in small Japanese families with idiopathic superior oblique muscle palsy [2,23]. In contrast, only two reports have described genome-wide association studies of Caucasian populations with esotropia [33] and self-reported strabismus of an unknown entity [34], respectively. These studies showed *GET1* (*WRB*) as a susceptibility gene for esotropia [33] and *TSPAN10* as one for self-reported strabismus [34]. In the present research, we conducted a genome-wide association study (GWAS) of Japanese patients with esotropia and exotropia of comitant strabismus as well as idiopathic superior oblique muscle palsy in comparison with three different Japanese cohorts: BBJ (180K) and BBJ (ASA) as two cohorts of BioBank Japan (BBJ), and also the Nagahama Cohort.

## 2. Results

### 2.1. Eligible Case Samples

A call rate equal to or greater than 0.99 was obtained in 711 of 723 case samples, which passed the quality check for genomic DNA. SNPs which did not fit for analysis were then deleted based on the following parameters and cutoff values: the mean normalized intensity (R Mean) (AA, AB, and BB) < 0.25, where AB indicated the heterozygote cluster, and cluster separation (Cluster Sep) < 0.4 for SNPs on the Y chromosome; or the R Mean (AA, AB, and BB) < 0.25 and Cluster Sep < 0.4, and call frequency (Call Freq) < 0.99 on the other chromosomes except for the Y chromosome. The number of SNPs determined was 657,060 on the Infinium Asian Screening Array-24 v.1.0 BeadChip.

Of the 711 patients with quality checks, 253 patients had esotropia, 356 had exotropia, and 102 had idiopathic superior oblique muscle palsy. Among the 253 patients with esotropia, infantile esotropia was diagnosed in 84 patients, accommodative esotropia including partially accommodative esotropia in 24, acute-onset (acquired) esotropia in 6, and esotropia (unclassified) in 139. In 356 patients with exotropia, intermittent exotropia was diagnosed in 131 patients, and constant exotropia in 225 patients.

**Figure 1:**
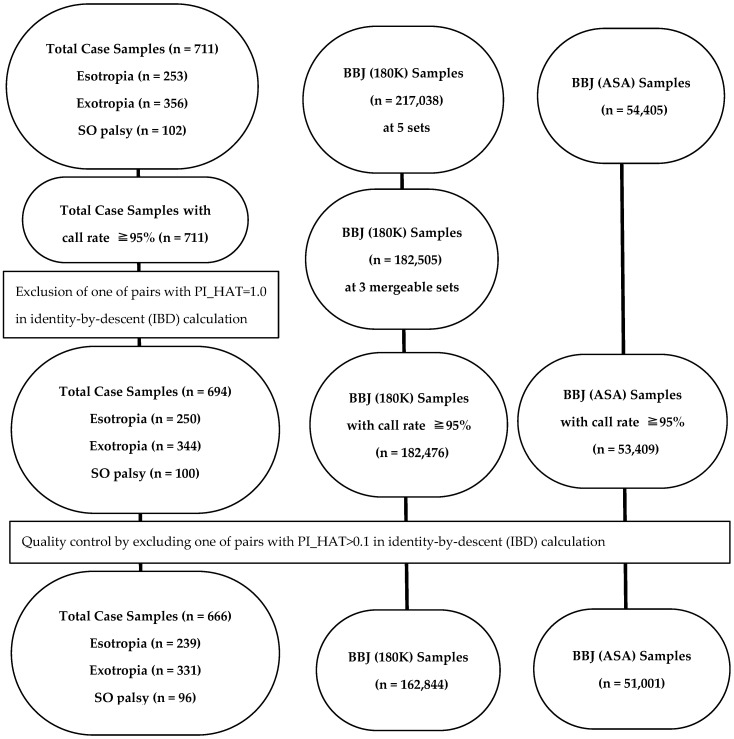
Flow charts for selection process of case samples and control samples in BBJ (180K) and BBJ (ASA). BBJ, BioBank Japan; SO palsy, idiopathic superior oblique muscle palsy.

**Figure 2:**
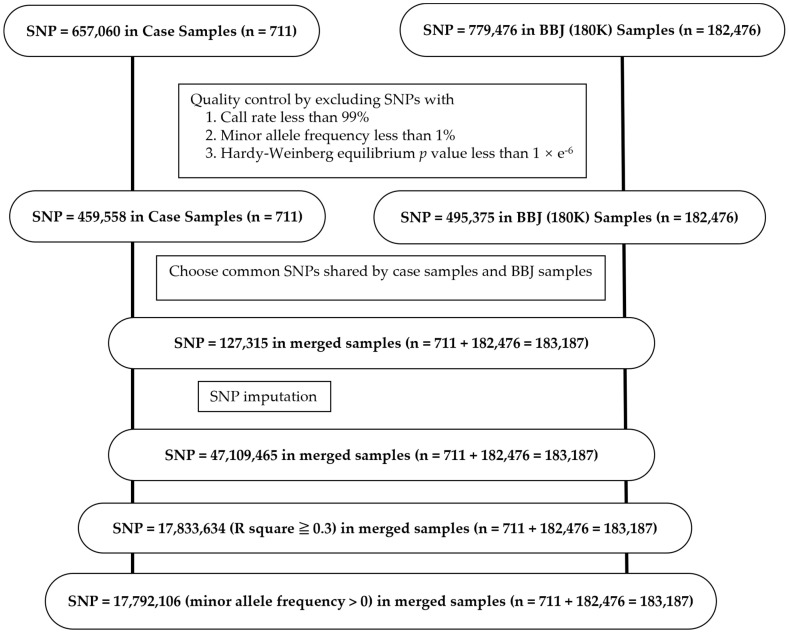
SNP selection and SNP imputation in case samples and control samples in BBJ (180K). BBJ, BioBank Japan.

**Figure 3:**
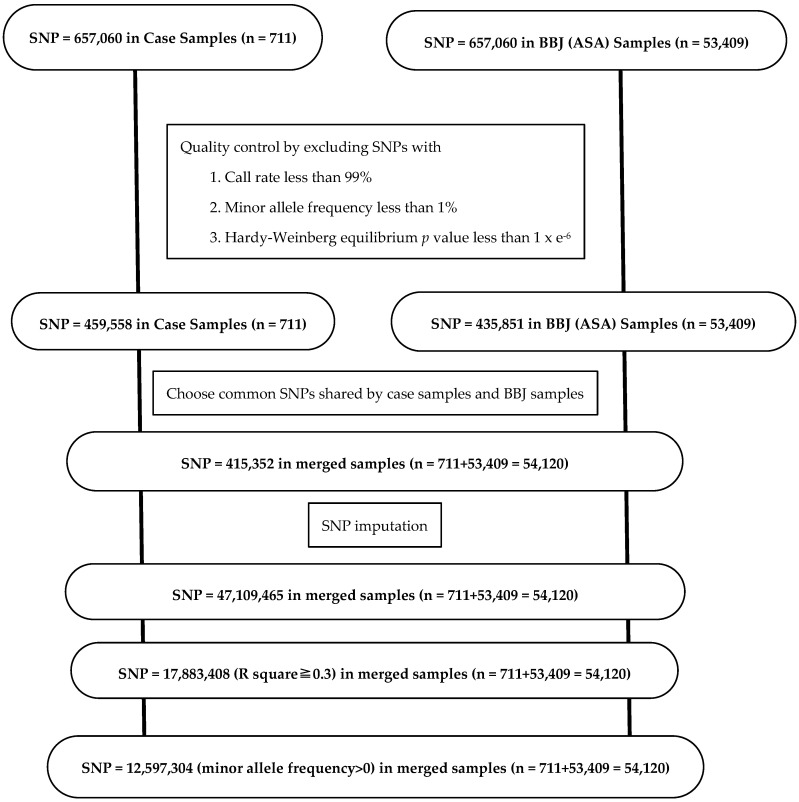
SNP selection and SNP imputation in case samples and control samples in BBJ (ASA). BBJ, BioBank Japan.

**Figure 4:**
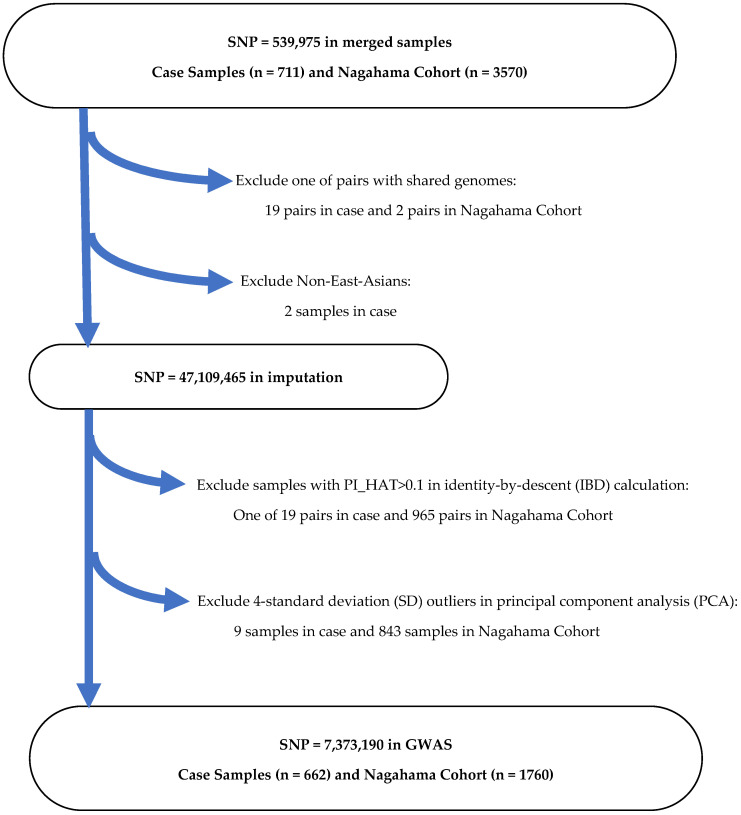
SNP selection and SNP imputation in case samples and control samples in Nagahama Cohort.

In identity-by-descent (IBD) estimation in the 711 patients (Figure 1), 20 pairs (8 pairs with esotropia, 9 pairs with exotropia, and 3 pairs with idiopathic superior oblique muscle palsy) showed PI_HAT = 0.5, while 19 pairs (3 pairs with esotropia, 12 pairs with exotropia, 2 pairs with esotropia in one individual and exotropia in the other, and 2 pairs with idiopathic superior oblique muscle palsy) showed PI_HAT = 1. After the exclusion of patients with PI_HAT > 0.1, 666 case samples in a GWAS with BBJ (180K) (Figure 2) or BBJ (ASA) (Figure 3) consisted of 239 patients with esotropia, 331 patients with exotropia, and 96 patients with idiopathic superior oblique muscle palsy (Figure 1). In a GWAS with the Nagahama Cohort, the number of case samples was 662 after the different flow of exclusion (Figure 4).

### 2.2. Imputed SNP Dosage in Three Different Control Cohorts

**Figure 5:**
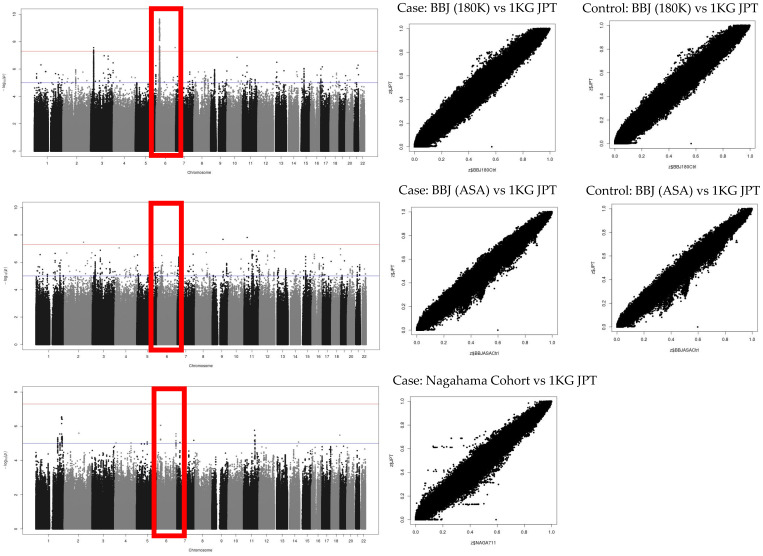
Comparison of SNP dosage after SNP imputation. Note that there is no difference between 1KG JPT in the vertical axis and imputed SNPs in the horizontal axis. SNPs in chromosome 6 (red box) were used as shown in Manhattan plot where red line indicates *p* = 5 × 10^−8^ and blue line indicates *p* = 1 × 10^−6^. Upper panels: GWAS with BBJ (180K). Middle panels: GWAS with BBJ (ASA). Lower panels: GWAS with Nagahama Cohort. 1KG JPT, Japanese population of 1000 Genomes Project.

**Figure 6:**
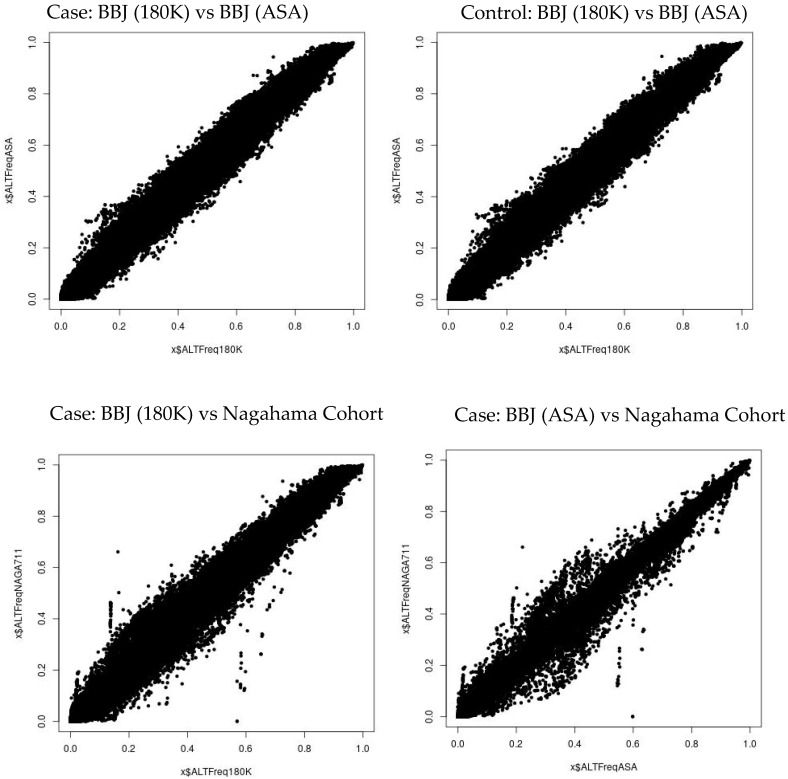
Comparison of SNP dosage after SNP imputation. Note that there is no difference between BBJ (180K) and BBJ (ASA) in the case (**top left panel**) and the control (**top right panel**). Furthermore, note that there is no difference between BBJ (180K) and Nagahama Cohort (**bottom left panel**) and between BBJ (ASA) and Nagahama Cohort (**bottom right panel**) in the case.

The imputed SNP dosages in three control cohorts were compared with the SNP dosage in the Japanese population (1KG JPT) of the 1000 Genomes Project to show no deviation in allele frequencies (Figure 5). The imputed SNP dosages were also compared among the three case–control groups to show no deviation in allele frequencies (Figure 6).

### 2.3. GWAS with Three Different Control Cohorts

**Figure 7:**
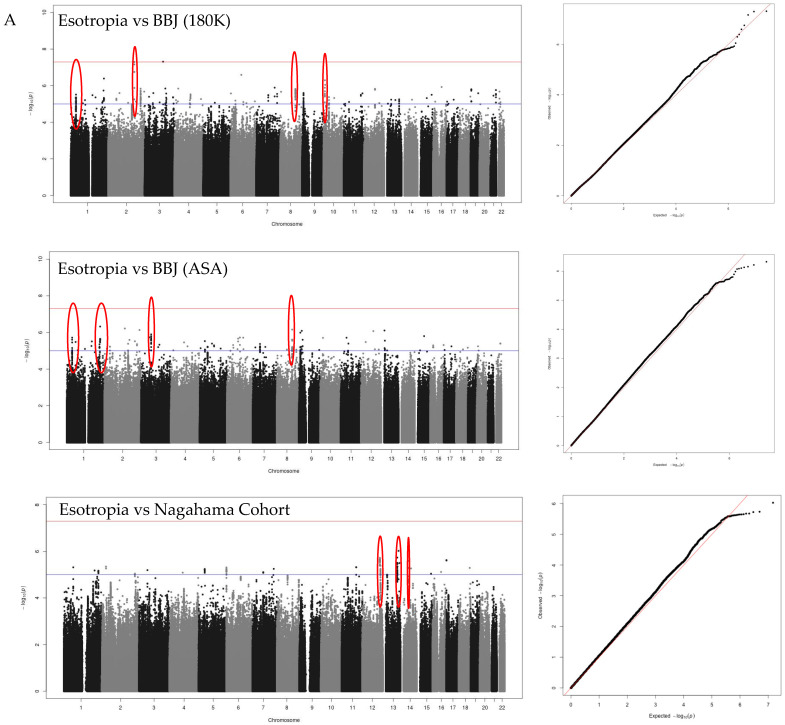

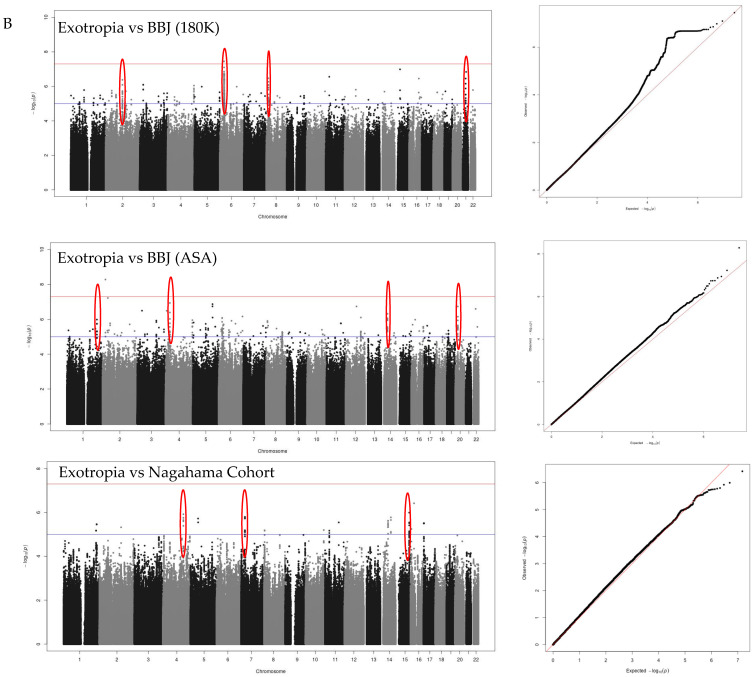

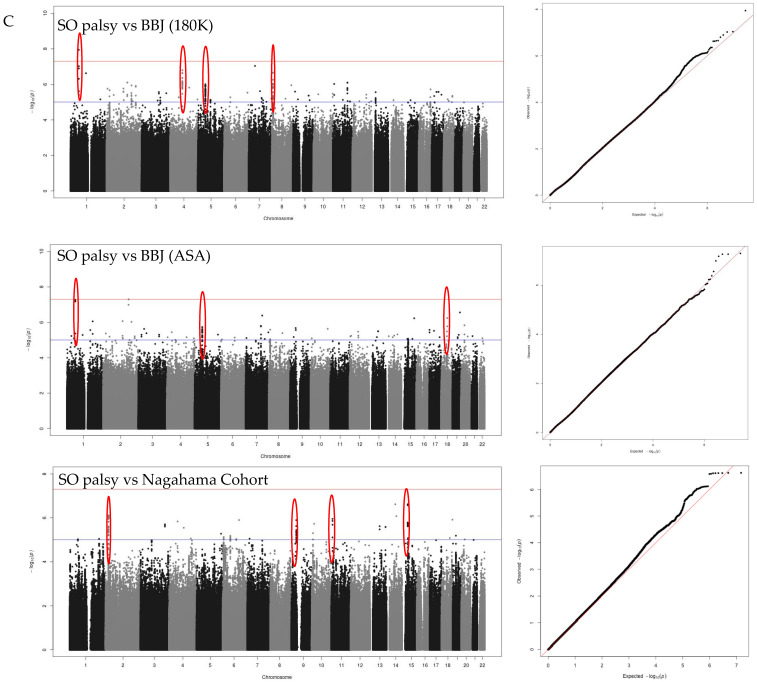

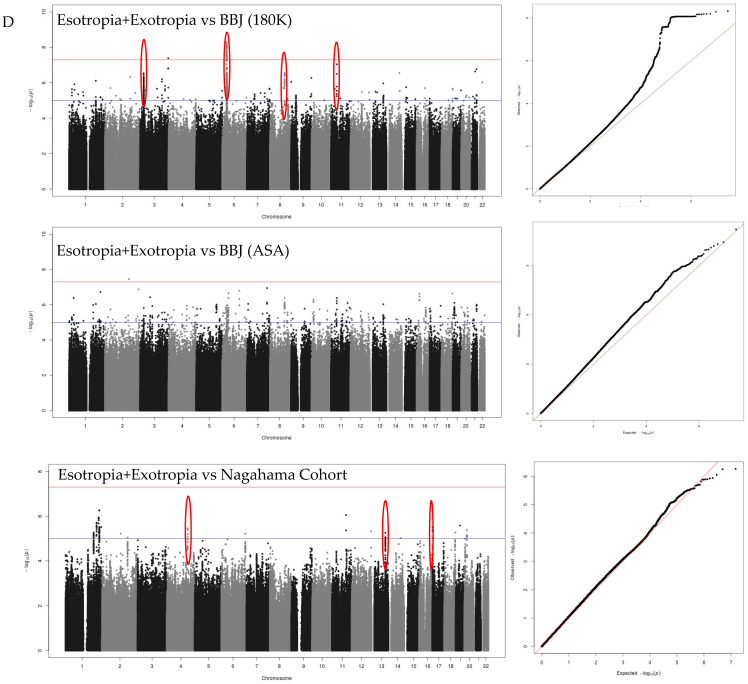

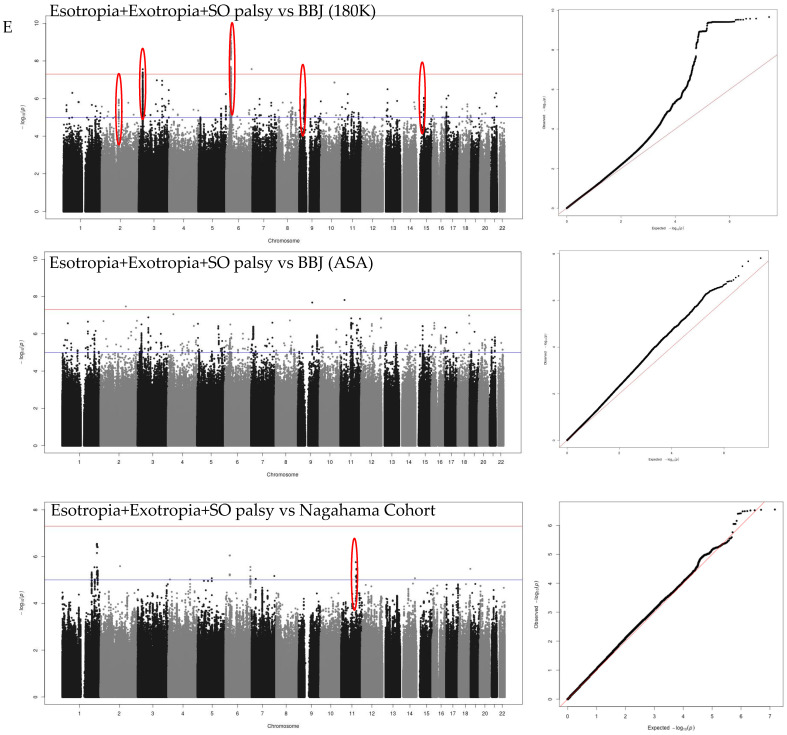
Meaningful peaks (red circles) formed by proximal SNPs in each Manhattan plot with control cohort of BBJ (180K), BBJ (ASA), and Nagahama. Chromosomal location on the horizontal axis versus −log_10_(*p*) on the vertical axis of each Manhattan plot. Red line indicates *p* = 5 × 10^−8^ and blue line indicates *p* = 1 × 10^−6^. Q–Q (log quantile–quantile *p*-value) plots with expected −log_10_(*p*) on the horizontal axis versus observed −log_10_(*p*) on the vertical axis, shown next to each Manhattan plot. Red line in each Q–Q plot indicate a correlation line. Only the group of exotropia is suggested to have a structured bias from the Q–Q plots. The case group is esotropia (**A**), exotropia (**B**), idiopathic superior oblique muscle palsy (**C**), comitant strabismus of esotropia and exotropia (**D**), and all cases of esotropia, exotropia, and idiopathic superior oblique muscle palsy (**E**).

Manhattan plots were drawn with chromosomal location on the horizontal axis and statistical value, −log_10_(*p*-value), on the vertical axis for esotropia (Figure 7A), exotropia (Figure 7B), idiopathic superior oblique muscle palsy (Figure 7C), the combination of esotropia and exotropia (Figure 7D), and all entities of esotropia, exotropia, and superior oblique muscle palsy (Figure 7E). In three series of GWASs with three different control cohorts, meaningful peaks with a loose standard of *p* < 1 × 10^−6^ formed by the gathering of proximal SNPs in the linkage are indicated by red circles in each plot. Q–Q (log quantile–quantile *p*-value) plots which were depicted with the expected −log_10_(*p*) on the horizontal axis versus the observed −log_10_(*p*) on the vertical axis are shown next to each Manhattan plot. Only the group of exotropia was suggested to have a structured bias from the Q–Q plots.

**Figure 8:**
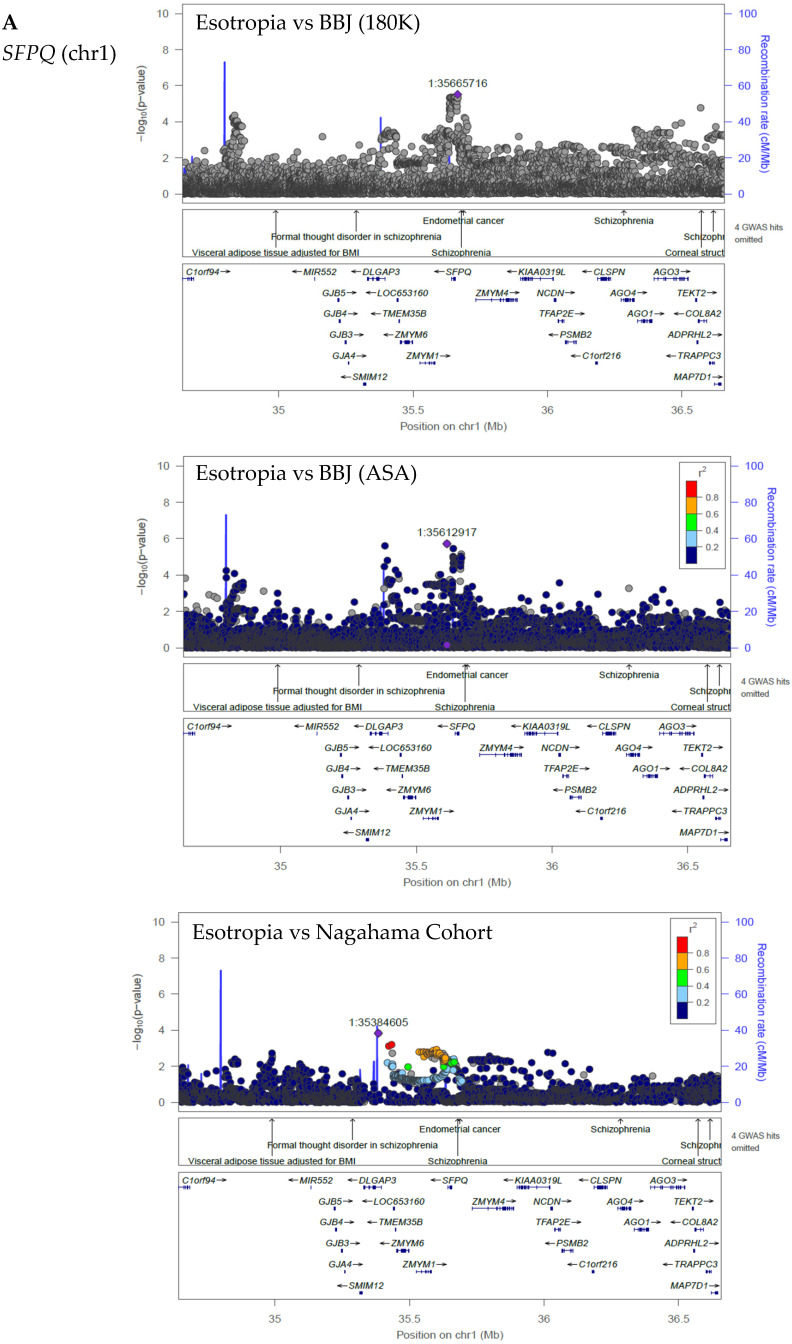

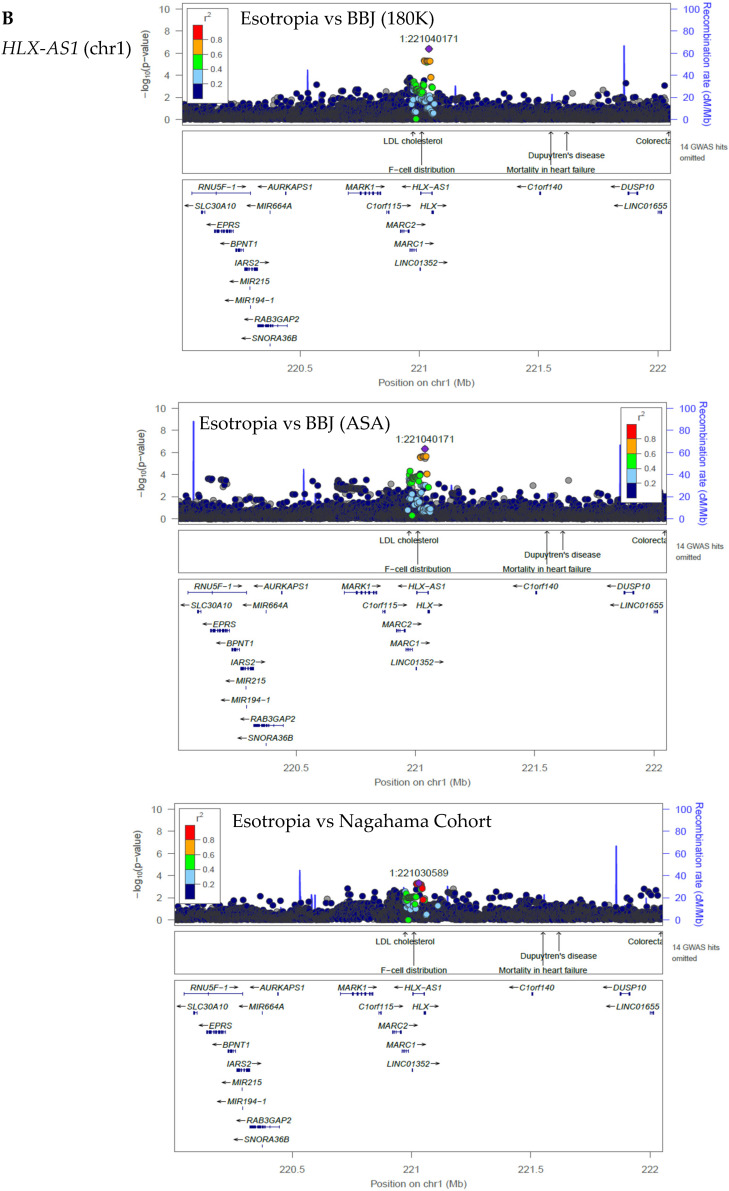

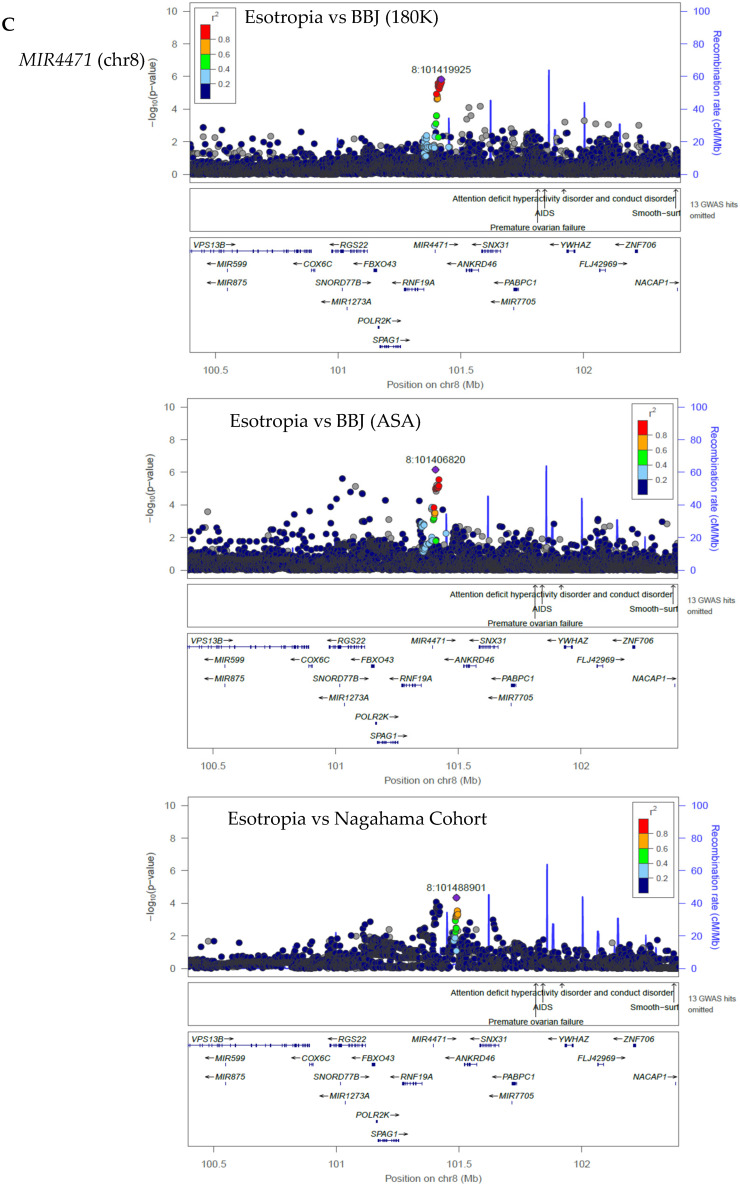

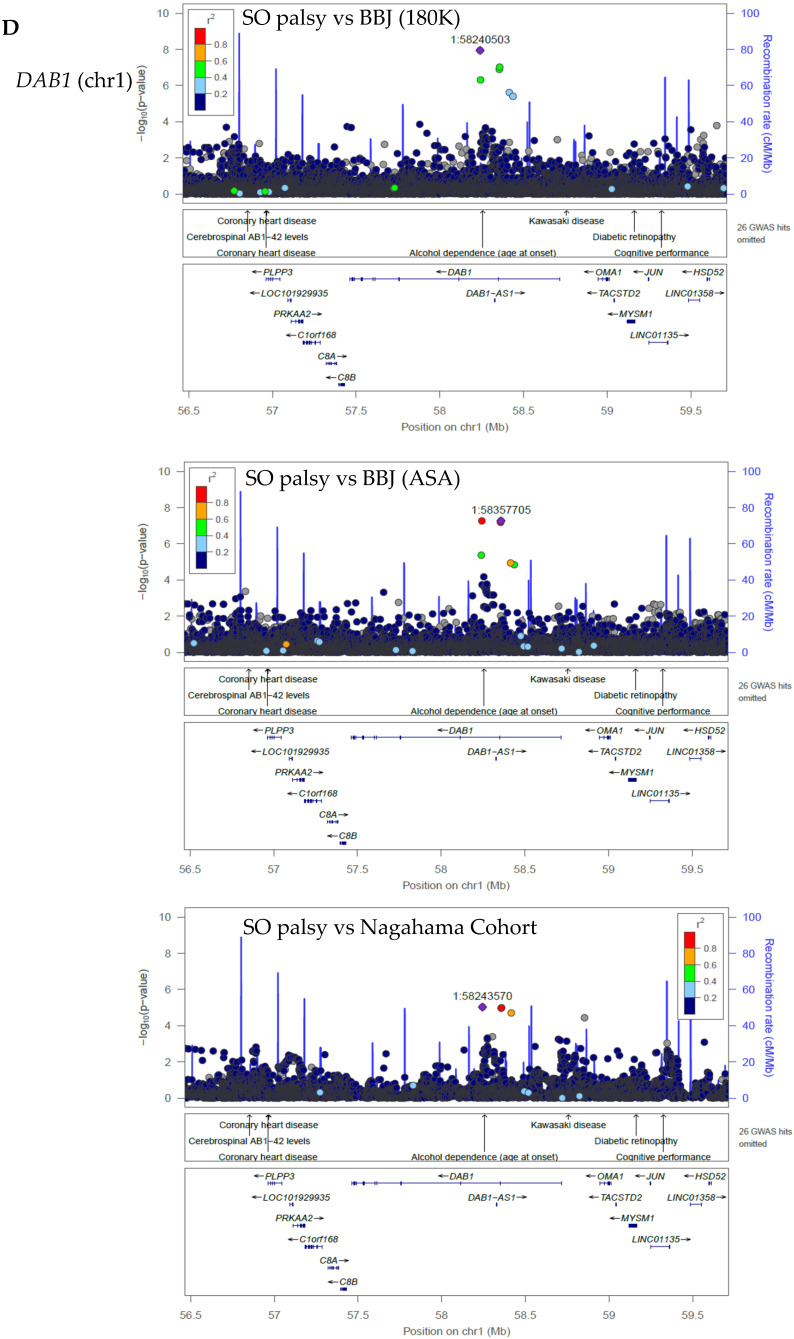

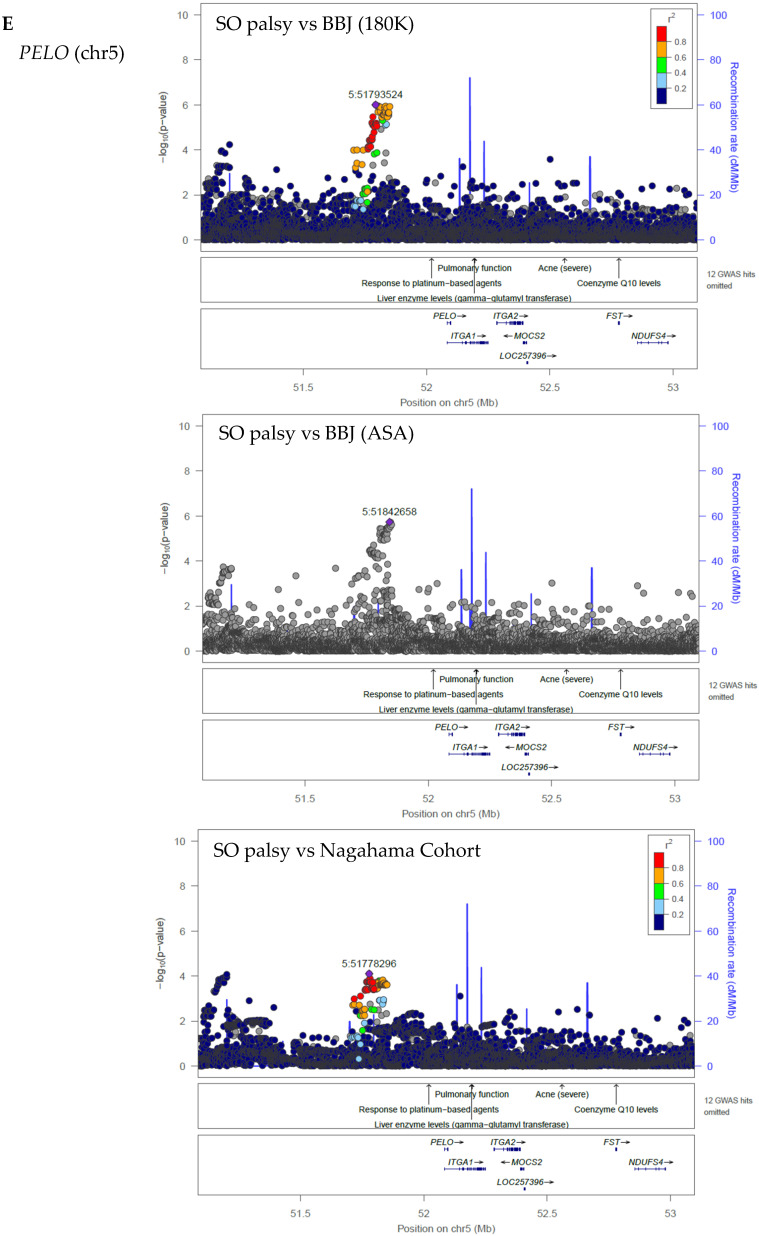
Regional plots by Locus Zoom of common peaks with a loose standard of *p* < 1 × 10^−6^ in 3 series of GWASs with 3 different control cohorts of BBJ (180K), BBJ (ASA), and Nagahama Cohort. The case group is esotropia (**A**–**C**) and idiopathic superior oblique muscle palsy (**D**,**E**).

As common peaks in three series of GWASs with three different control cohorts, three loci were suggested for esotropia (*SFPQ* and *HLX-AS1* in chromosome 1, and *MIR4471* in chromosome 8 as candidate genes; Figure 8A–C, Table 1) and two loci for idiopathic superior oblique muscle palsy (*DAB1* in chromosome 1 and *PELO* in chromosome 5 as candidate genes; Figure 8D,E, Table 1).

At the standard significance of *p* < 5 × 10^−8^ (Table 2, Supplementary Figure S1), *CDCA7* in chromosome 2 for esotropia, *HLA-F* in chromosome 6 for exotropia, and *DAB1-AS1* in chromosome 1 for idiopathic superior oblique muscle palsy were suggested as candidates in a GWAS with BBJ (180K). Additionally, in a GWAS with BBJ (180K), *ACAP2* in chromosome 3 and *HLA-F* in chromosome 6 were suggested as candidates in the combined group of esotropia and exotropia. In a GWAS with BBJ (180K), *RARB* in chromosome 3 and *HLA-F* in chromosome 6 were suggested as candidates in the combined three-entities group with esotropia, exotropia, and idiopathic superior oblique muscle palsy. In contrast, no peak with *p* < 5 × 10^−8^ was found in a GWAS with BBJ (ASA) or the Nagahama Cohort.

## 3. Discussion

The long-term goal of our studies up until now is to search for strabismus susceptibility genes. In our initial attempt, we used linkage analyses in small-sized Japanese families with comitant strabismus including esotropia and exotropia [28,29]. In our previous study of linkage analyses, we have reached two potential candidate genes for strabismus susceptibility, *MGST2* and *WNT2* in chromosomal loci 4q28.3 and 7q31.2, respectively [32]. We then proceeded to make *MGST2*-deficient mice by CRISPR-Cas9 technology and proved that eyeballs in the homozygous mice were in an ovoid shape and significantly larger in size and volume than the wild-type eyeballs, by measurements with magnetic resonance imaging [35]. The changes in the shape and size of the eyeballs might result in a different torque of the extraocular muscles which attach to the surface of the eyeballs, and, thus, might lead to the different alignment of both eyes.

Since the alignment of both eyes would be influenced by the innervation of the extraocular muscles with ocular motor nerves such as the oculomotor, trochlear, and abducens nerves from the brainstem, candidate genes for strabismus susceptibility might be expressed in the brain and have an effect on the function of the brain. Furthermore, binocularity such as stereopsis belongs to a higher function of the central nervous system to integrate visual sensory input from both eyes. In this context, we had also carried out whole-genome sequencing in three small families with idiopathic superior oblique muscle palsy as a major form of non-comitant strabismus [2]. In that previous study, we combined the two methods of linkage analysis and SNP sorting, and we reached several candidate genes for idiopathic superior oblique muscle palsy [2].

Based on the rationale that a different approach in genetic statistics would help reach candidate genes for strabismus susceptibility, we performed a series of the genome-wide association study (GWAS) for esotropia and exotropia of comitant strabismus as well as idiopathic superior oblique muscle palsy in the entity of non-comitant strabismus in the present research. A critical point in the GWAS is what kind of a genetic cohort should be used as a control. We first chose the preexisting BBJ cohort of the Japanese population which was genotyped by a different array from that used in strabismic patients in this study. In the condition that different arrays are used separately to genotype the case and control, the set of loaded SNPs in each array was not exactly the same. Therefore, the common SNPs between the different arrays have to be chosen and the number of SNPs used for a comparison of the case with the control is reduced to a large extent. SNP imputation based on the smaller number of common SNPs would be less accurate. The cohort in the Tohoku Medical Megabank (ToMMo) which was typed by the same Asian array was not used in this study since the population in the northern part of Japan is somewhat different from that in the western part of Japan where the patients in this study resided. Therefore, we chose the cohort of the Nagahama Study with participants living in western Japan, although the number is small. Meanwhile, BBJ released a new series of a cohort of the Japanese population which was genotyped by the Asian array.

In the GWAS, related individuals are excluded from the case population and control population, based on the identity-by-descent (IBD) analysis. In an attempt to test different conditions in statistical analyses, we initially excluded only one of the pairs with IBD = 1.0 in the case cohorts and retained pairs with IBD = 0.5 to perform the GWAS since the case samples were collected not only from independent individuals but also from family members with strabismus for the linkage analysis [28,29,32]. We also performed a GWAS with the case samples with the exclusion of IBD > 0.1 in the usual setting. The GWAS results were basically the same whether the case samples did or did not contain pairs with IBD = 0.5.

In the case groups, patients with esotropia, exotropia, or idiopathic superior oblique muscle palsy were defined as each independent group to perform a GWAS with three different control cohorts. In addition, we defined, as the case group, the combined group of patients with esotropia and exotropia of comitant strabismus, and also defined the all-combined samples with esotropia, exotropia, and idiopathic superior oblique muscle palsy. A rationale for combining the different entities of comitant and non-comitant strabismus is that these different phenotypes are indeed present in a pedigree [28,29,36]. A merit of combining patients with different phenotypes would be that the number of patients in a case group becomes larger, large enough to detect a significant locus which would serve as a common factor for comitant and non-comitant strabismus with a genetic background. In contrast, a demerit would be that the combined case group with heterogeneous phenotypes would dilute the significant locus which would be specific for each group with different phenotypes.

The Nagahama Cohort genotyped by the same Asian array which was used for the genotyping of the case group had too small a number of individuals to detect significant loci. The GWAS using BBJ (ASA) as a control cohort could not detect significant loci even though this control cohort had a large number of individuals and shared the same typing array with the case group in this study. In contrast, the GWAS with the largest number of control individuals in BBJ (180K) in this study did reach strabismus susceptibility loci at the significant level of *p* = 5 × 10^−8^. Since the typing arrays were different between the case group and the control group of BBJ (180K), common SNPs were chosen first to proceed to SNP imputation. Therefore, the SNP imputation based on the smaller number of SNPs in the GWAS with BBJ (180K) would be less accurate than the GWAS with BBJ (ASA). Under the circumstances, we tried to find common significant loci which were shared by three series of GWASs with three different sets of control cohorts. In order not to miss any interested loci, the significant level was loosened to *p* = 1 × 10^−6^ from the standard level of *p* = 5 × 10^−8^ in this research attempt. This approach would be expected to reinforce the findings in a single GWAS with BBJ (180K) which was genotyped by the different array.

The loci for strabismus susceptibility which were detected as significant in this GWAS did not overlap with the loci which were reported in previous linkage analyses [25,26,27,28,29,30,31,32,36] and a preceding GWAS in other populations [33,34]. *MGST2* (microsomal glutathione S-transferase 2 at 4q31.1) was detected as a susceptibility gene for comitant strabismus of esotropia and exotropia by our previous linkage study [32] but was not replicated by the present GWAS. *WRB* (tryptophan-rich basic protein, also known as *GET1*, guided entry of tail-anchored proteins factor 1, at 21q22.2) and *TSPAN10* (tetraspanin 10 at 17q25.3), which were detected as a susceptibility gene for esotropia in the U.S.A. [33] and for self-reported strabismus of unknown entity in the U.K. [34], respectively, were also not replicated by the present GWAS (see regional plots in Supplementary Figure S2). Since strabismus is a multifactorial disease, the loci detected in this GWAS and the other loci detected by the preceding linkage studies and GWAS would play a role in the development of strabismus like polygenetic risk scores which have been described in GWASs for other diseases such as diabetes mellitus [37,38,39,40]. As strabismus susceptibility genes, this GWAS suggests non-coding RNAs, which have been also suggested by the other studies [41,42].

Notably, *DAB1* was detected as a susceptibility gene at a significant level of *p* < 5 × 10^−8^ for idiopathic superior oblique muscle palsy in the GWAS with BBJ (180K), and was also detected as such at a weak level of significance of *p* < 1 × 10^−6^ in the GWAS with BBJ (ASA) and the Nagahama Cohort (Table 1 and Table 2). *DAB1* is involved in neuronal migration and is a responsible gene for spinocerebellar ataxia type 37 [43]. Trochlear nerve hypoplasia is considered to be a cause for idiopathic superior oblique muscle palsy which manifests as varying levels of the hypoplasia of the superior oblique muscle [2,17,19,20]. Under the circumstances, aberrant neuronal migration would serve as the background for the trochlear nerve hypoplasia [41]. In comparison with the control cohort, BBJ (180K), *RARB* (retinoic acid receptor-β) was detected as a candidate at a significant level of *p* < 5 × 10^−8^ in the combined group of esotropia, exotropia, and idiopathic superior oblique muscle palsy (Table 2). Since retinoic acids are involved in neural crest cell migration in eye morphogenesis [44,45,46], retinoic acids would have a role in the development of extraocular muscles of neural crest cell origin [47,48].

A major limitation in this study was that the three control cohorts used in this study would contain unidentified individuals with strabismus, although in a small number. This limitation would be cancelled by the large number of individuals in the control cohorts, compared with the case cohort. Another limitation is the fact that clinical manifestations as phenotypes vary largely from individual to individual in each entity of esotropia, exotropia, and idiopathic superior oblique muscle palsy. For instance, the degree of deviation is different and the level of stereopsis is different from individual to individual. The diagnosis of each entity in the case samples was, of course, made by ophthalmologists with a subspecialty in strabismus, based on the diagnostic criteria. Interestingly, a candidate gene, *DAB1*, was detected reproducibly in idiopathic superior oblique muscle palsy by the series of GWASs with all three control cohorts. The results suggest the entity of idiopathic superior oblique muscle palsy would be more homogeneous, compared with the entities of esotropia and exotropia, from the viewpoint of the diagnostic criteria.

In conclusion, this study is the first to conduct a GWAS for comitant and non-comitant (paralytic) strabismus in the Japanese population. To the best of our knowledge, this study is the first, especially, to conduct a GWAS for idiopathic superior oblique muscle palsy. The series of GWASs with three different control cohorts would be an effective method to search for candidate genes for multifactorial diseases such as strabismus. The susceptibility genes detected in this study remain candidates, and, thus, future studies will be required for the functional validation of these candidate genes in comitant and non-comitant strabismus.

## 4. Materials and Methods

### 4.1. Participants

Participants in this study were patients diagnosed with esotropia or exotropia of comitant strabismus, or idiopathic superior oblique muscle palsy at Okayama University Hospital (Figure 1). Genomic DNA was isolated from leukocytes of 10 mL peripheral blood in each patient after written consent to be involved in the study. The study conformed to the tenets of the Declaration of Helsinki and was approved by the Ethics Committee of Okayama University Graduate School of Medicine, Dentistry, and Pharmaceutical Sciences and Okayama University Hospital (Identifier: 1512-027, date of approval on updated version: 14 August 2020).

### 4.2. Genotyping of Participants

Genome-wide SNP genotyping was carried out with Infinium Asian Screening Array-24 v.1.0 BeadChip Kit (Illumina, San Diego, CA, USA) at Riken Genesis, Co., Ltd. (Tokyo, Japan). The quality of genomic DNA was assessed by agarose gel electrophoresis and the PicoGreen assay (Thermo Fisher Scientific, Waltham, MA, USA) using a plate reader (SpectraMax, Molecular Devices, San Hose, CA, USA). Whole genome of each sample which passed the quality check was alkali-denatured, neutralized, and amplified enzymatically by Multi-Sample Amplification Master Mix (MSM, Illumina) overnight in an incubation oven (Illumina). The amplified DNA was enzymatically fragmented by Fragmentation Solution (FMS, Illumina) for one hour in a Hybex microsample incubator (SciGene, Sunnyvale, CA, USA). The fragmented DNA was precipitated with 2-propanol by centrifugation, dissolved in hybridization buffer, and denatured at 48 °C overnight in a hybridization oven (Illumina). The fragmented and denatured DNA in a microsample incubator was kept at 95 °C for 20 min, and, then, was dispensed with Tecan system (Maennedorf, Switzerland) to the BeadChip, and hybridized at 48 °C overnight in the hybridization oven. Unhybridized free DNA on the BeadChip was washed away, and primers which hybridized with the sample DNA were extended with labelled nucleotides by Extension Mix Long (EML, Illumina). The BeadChip was coated with an anti-decay reagent, dried in a vacuum dessicator, and placed on a carrier of iScan system (Illumina) for scan.

The imaging data were analyzed by genotyping analysis software GenomeStudio 2011.1 (Illumina) to calculate the call rate of each sample. Data were built at 4 steps in GenomeStudio: at step 1 to use standard cluster file before quality check, at step 2 to perform quality check to delete samples with low call rates, at step 3 to perform cluster optimization, and at step 4 to perform SNP quality check by parameter-setting to delete SNPs which did not fit for analysis. Clustering Algorithm was set at GenTrain 2.0 and GenCall Threshold was set at 0.15 in GenomeStudio. Genotyping data output was carried out by the software GenomeStudio Framework version 2013 and Genotyping Module version 2.0.3.

### 4.3. Control Samples

Three Japanese cohorts were used as control samples in genome-wide association study, and were designated in this study as BBJ (180K), BBJ (ASA), and Nagahama. In the first cohort (Figure 1), BBJ (180K), the SNP data of 217,038 samples in BioBank Japan which were registered as 47 target common diseases [49] were obtained from the National Bioscience Database Center (NBDC) after ethical approval (Identifier: 0016, date of approval: 15 February 2019) by the Institutional Review Board affiliated with Tsukuba International Clinical Pharmacology Clinic (Tsukuba City, Japan) for the use as control, based on the Ethical Guidelines for Medical and Health Research Involving Human Subjects, issued by the Government of Japan. These BioBank Japan data were genotyped by Illumina Infinium OmniExpressExome-8 v1.0 or v1.2 or Illumina HumanOmniExpress-12 v1.0. Samples with call rate less than 95% were excluded to obtain dataset of 182,476 samples for analysis.

In the second cohort (Figure 1), BBJ (ASA), genome-wide SNP data of 54,405 samples were obtained from NBDC after ethical approval (Identifier: 0026, date of approval: 28 March 2022) by the Institutional Review Board affiliated with Tsukuba International Clinical Pharmacology Clinic (Tsukuba City, Japan) for the use as control. The genotype data were determined by Infinium Asian Screening Array-24 v1.0 BeadChip Kit in 11,716 individuals from BBJ first cohort and 42,689 individuals from BBJ second cohort (Dataset ID JGAS000412, Release Date: 30 November 2021). The controlled access of the dataset was approved as Data in Use ID JGAD000529 (Principal Investigator: Shigeo Kamitsuji, Affiliation: Statistical Analysis Division, Stagen Co., Ltd., Country/Region: Japan, Research Title: Genome-Wide Association Study to identify genetic factors for strabismus in Japanese population, Period of Data Use: 13 February 2023–28 February 2027). Samples with call rate less than 95% were excluded to obtain dataset of 53,409 samples for analysis.

In the third cohort, Nagahama, the genotype data were determined by Infinium Asian Screening Array-24 v1.0 BeadChip Kit in 3570 individuals who resided in Nagahama City, Shiga Prefecture in western Japan. The project is a collaboration between Kyoto University and Nagahama City [50] and this study was approved by Ethics Committee in Kyoto University (Identifier: G1289, date of approval: 26 November 2020).

### 4.4. Whole-Genome Imputation

Quality control of SNPs was carried out by allele frequency and R-square value. In the case group, expected minimum allele frequency was defined as 1/(the number of cases × 2). In the control group, the minimum allele frequency was evaluated by 95% confidence interval of alternative allele frequency in the 1000 Genomes Project JPT (Japanese Population in Tokyo) [51,52]. The fitness with Hardy–Weinberg equilibrium principle was evaluated by R-square values ≥ 0.3 which were used for quality control in SNP imputation. Cryptic relatedness of samples to each other was estimated by identity-by-descent (IBD) PI_HAT values in plink [53].

Based on quality control to exclude SNPs with call rate less than 99%, minor allele frequency less than 1%, and *p*-value of Hardy–Weinberg equilibrium test less than 1 × 10^−6^, 459,558 of 657,060 SNPs were chosen in 711 samples of comitant strabismus or idiopathic superior oblique muscle palsy (Figure 2). According to the same standard, 495,375 of 779,476 SNPs were chosen in 182,476 samples of the BBJ (180K). Based on the reference panels for Japanese population [51,52], imputation was carried out by the software Beagle v5.1 and plink2 [53] in 1269 of 1304 chunks with 2 Mbp length for the total 127,315 SNPs which were common between the case and the control [54,55,56,57]. The remaining chunks could not be imputed because of no SNP data on those chunks. The data of 17,792,106 SNPs were used for genome-wide association study after quality control of 47,109,465 imputed SNPs by imputation quality metric R-square (estimated r^2^, specific to each SNP) equal to or greater than 0.3 and minor allele frequency greater than zero (Figure 2).

In the case of BBJ (ASA) as control samples, 435,851 of 657,060 SNPs were chosen in 53,409 samples of BBJ (ASA), according to the same standard as described above. Based on the reference panels for Japanese population [51,52], imputation was carried out by a method of pre-phasing imputation with Minimac4 for 415,352 SNPs which were common between the case and the control [58,59,60]. The data of 17,883,408 SNPs were used for genome-wide association study after quality control of 47,109,465 imputed SNPs by imputation quality metric R-square (estimated r^2^, specific to each SNP) equal to or greater than 0.3. In GWAS, the number of SNPs was 12,597,304 after the final exclusion of 5,286,104 SNPs with minor allele frequency = 0 (Figure 3).

In the case of Nagahama Cohort as control samples, SNP imputation was carried out as shown in the flow chart (Figure 4).

### 4.5. Genome-Wide Association Study

As SNP dosage, the genotype value was defined as an expected value (continuous value from 0 to 2) for having the number of the alternative allele against the reference allele on the UCSC hg19 Genome Browser. Odds ratio for each SNP was estimated by one increment in SNP dosage in the case group, compared with the control group. The association was evaluated by Wald test and the distribution of *p*-values were depicted as a Manhattan plot. In logistic regression analysis, case group or control group was defined as an explanatory variable and SNP dosage as a response variable. When regression coefficient (BETA) is a positive value, the case group was interpreted to have the alternative allele (target allele in population) at a higher rate compared with the control group. Odds ratio estimated by one increment in SNP dosage in the case group, compared with the control group, could be obtained by calculation of eBETA [61,62]. In addition to the program, plink2 [53], regenie [61] was used for calculation to reduce other biases in the combined analysis of genotypes and covariates. The chromosomal areas in interest were depicted by the software Locus Zoom js v0.12 [63].

## Figures and Tables

**Table 1:** Common peaks with a loose significant level at *p* < 1 × 10^−6^ in GWAS with three different control cohorts in Japanese population.

GWAS	SNP	Chromosomal Location	Genes	Full Name	Function
Esotropia vs. BBJ (180K) Esotropia vs. BBJ (ASA) Esotropia vs. Nagahama	35,665,716 35,612,917 35,384,605	1p34.3	*SFPQ*	Splicing factor proline and glutamine rich	Alternative mRNA splicing
Esotropia vs. BBJ (180K) Esotropia vs. BBJ (ASA) Esotropia vs. Nagahama	22,1040,171 22,1040,171 22,1030,589	1q41	*HLX-AS1*	HLX antisense RNA 1	
Esotropia vs. BBJ (180K) Esotropia vs. BBJ (ASA) Esotropia vs. Nagahama	101,419,925 101,406,820 101,488,901	8q22.2	*MIR4471*	microRNA 4471	Post-transcriptional regulation of gene expression
SO palsy vs. BBJ (180K) SO palsy vs. BBJ (ASA) SO palsy vs. Nagahama	58,240,503 58,357,705 58,243,570	1p32.2-p32.1	*DAB1*	DAB adaptor protein 1	Neuronal migration
SO palsy vs. BBJ (180K) SO palsy vs. BBJ (ASA) SO palsy vs. Nagahama	51,793,524 51,842658 51,778,296	5q11.2	*PELO*	Pelota mRNA surveillance and ribosome rescue factor	Cell cycle control

Cited from the database “gene” in National Center for Biotechnology Information (NCBI) in U.S.A. GWAS in condition of the exclusion of identity-by-descent (IBD) > 0.1 and no regenie prediction. BBJ, BioBank Japan; Nagahama, Nagahama Cohort; SO palsy, idiopathic superior oblique (SO) muscle palsy. See Figure 8 for regional plots corresponding to each locus and Supplementary Table S1 for original data.

**Table 2:** Peaks with a standard significant level at *p* < 5 × 10^−8^ in GWAS with the control cohort, BBJ (180K).

GWAS	SNP	Chromosomal Location	Genes	Full Name	Function
Esotropia vs. BBJ (180K)	174,265,779	2q31.1	*CDCA7*	Cell division cycle associated 7	Cell transformation
Exotropia vs. BBJ (180K)	29,677,877	6p22.1	*HLA-F*	Major histocompatibility complex, class I, F	
SO palsy vs. BBJ (180K)	58,240,503	1p32.2	*DAB1-AS1*	DAB1 antisense RNA 1	Neuronal migration
ET+XT vs. BBJ (180K)	195,019,926	3q29	*ACAP2*	ArfGAP with coiled-coil, ankyrin repeat, and PH domains 2	Actin filament-based process
ET+XT vs. BBJ (180K)	29,677,877	6p22.1	*HLA-F*	Major histocompatibility complex, class I, F	
ET+XT+SO vs. BBJ (180K)	25,228,113	3p24.2	*RARB*	Retinoic acid receptor beta	Embryonic morphogenesis, cell growth and differentiation
ET+XT+SO vs. BBJ (180K)	29,677,877	6p22.1	*HLA-F*	Major histocompatibility complex, class I, F	

Cited from the database “gene” in National Center for Biotechnology Information (NCBI) in U.S.A. GWAS in condition of the exclusion of identity-by-descent (IBD) > 0.1 and no regenie prediction. BBJ, BioBank Japan; SO palsy, idiopathic superior oblique (SO) muscle palsy; ET, esotropia; XT, exotropia. See Supplementary Figure S1 for regional plots corresponding to each locus.

## Data Availability

Additional data are available upon reasonable request from the corresponding author.

## Supplementary Materials

The following supporting information can be downloaded at: https://www.mdpi.com/article/10.3390/ijms25136986/s1.
