# Supplementary material for: Genome-Wide Association Study with Three Control Cohorts of Japanese Patients with Esotropia and Exotropia of Comitant Strabismus and Idiopathic Superior Oblique Muscle Palsy
Source: Int J Mol Sci. 2024 Jun 26;25(13):6986. doi: 10.3390/ijms25136986 (PMC11241339; doi:10.3390/ijms25136986)
Supplement: Supplementary file 1 [file ijms-25-06986-s001.zip › Supplementary Figure S1 with legend.pptx]

## Slide 1
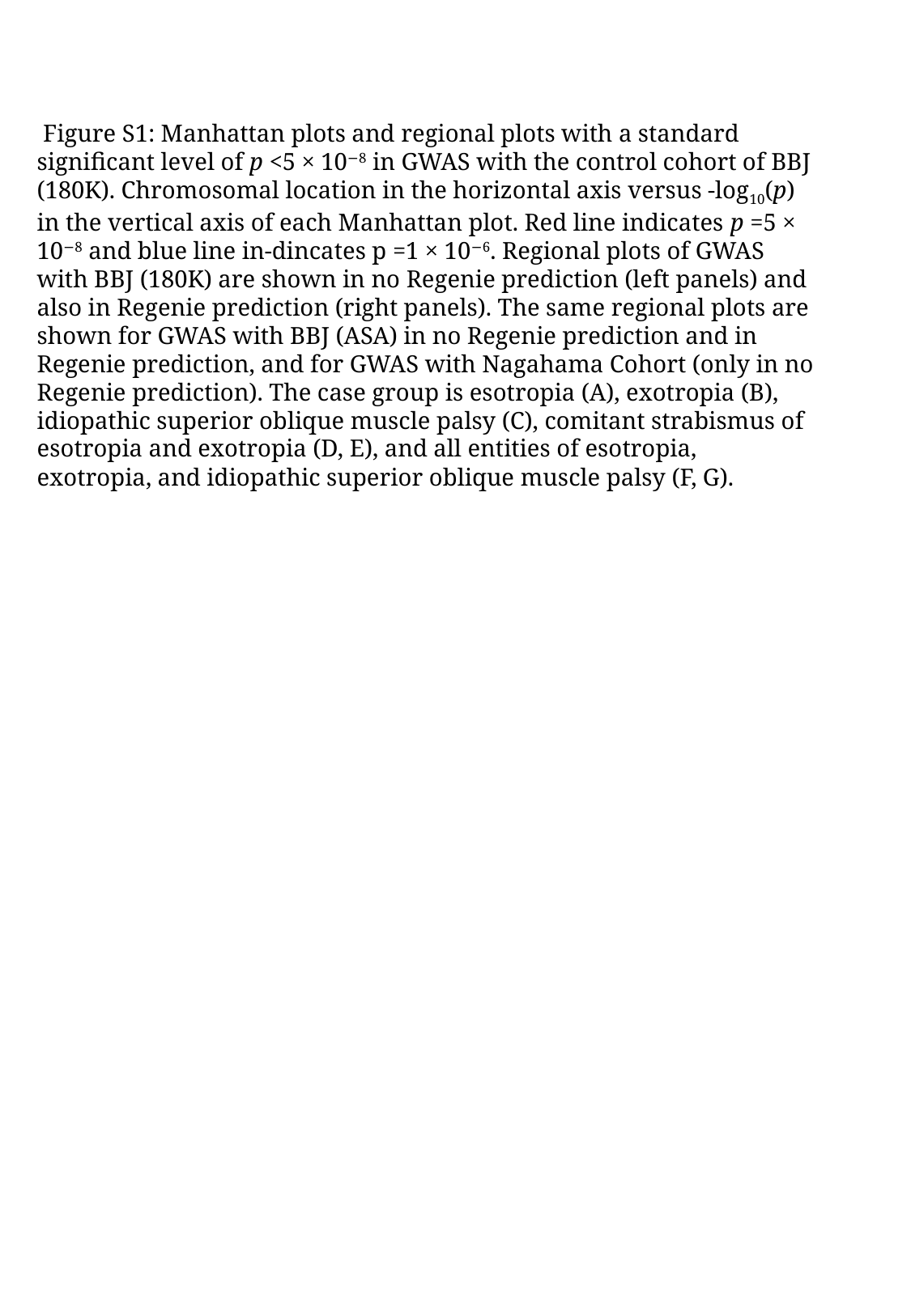

Figure S1: Manhattan plots and regional plots with a standard significant level of p <5 × 10^−8^ in GWAS with the control cohort of BBJ (180K). Chromosomal location in the horizontal axis versus -log10(p) in the vertical axis of each Manhattan plot. Red line indicates p =5 × 10^−8^ and blue line in-dincates p =1 × 10^−6^. Regional plots of GWAS with BBJ (180K) are shown in no Regenie prediction (left panels) and also in Regenie prediction (right panels). The same regional plots are shown for GWAS with BBJ (ASA) in no Regenie prediction and in Regenie prediction, and for GWAS with Nagahama Cohort (only in no Regenie prediction). The case group is esotropia (A), exotropia (B), idiopathic superior oblique muscle palsy (C), comitant strabismus of esotropia and exotropia (D, E), and all entities of esotropia, exotropia, and idiopathic superior oblique muscle palsy (F, G).

## Slide 2
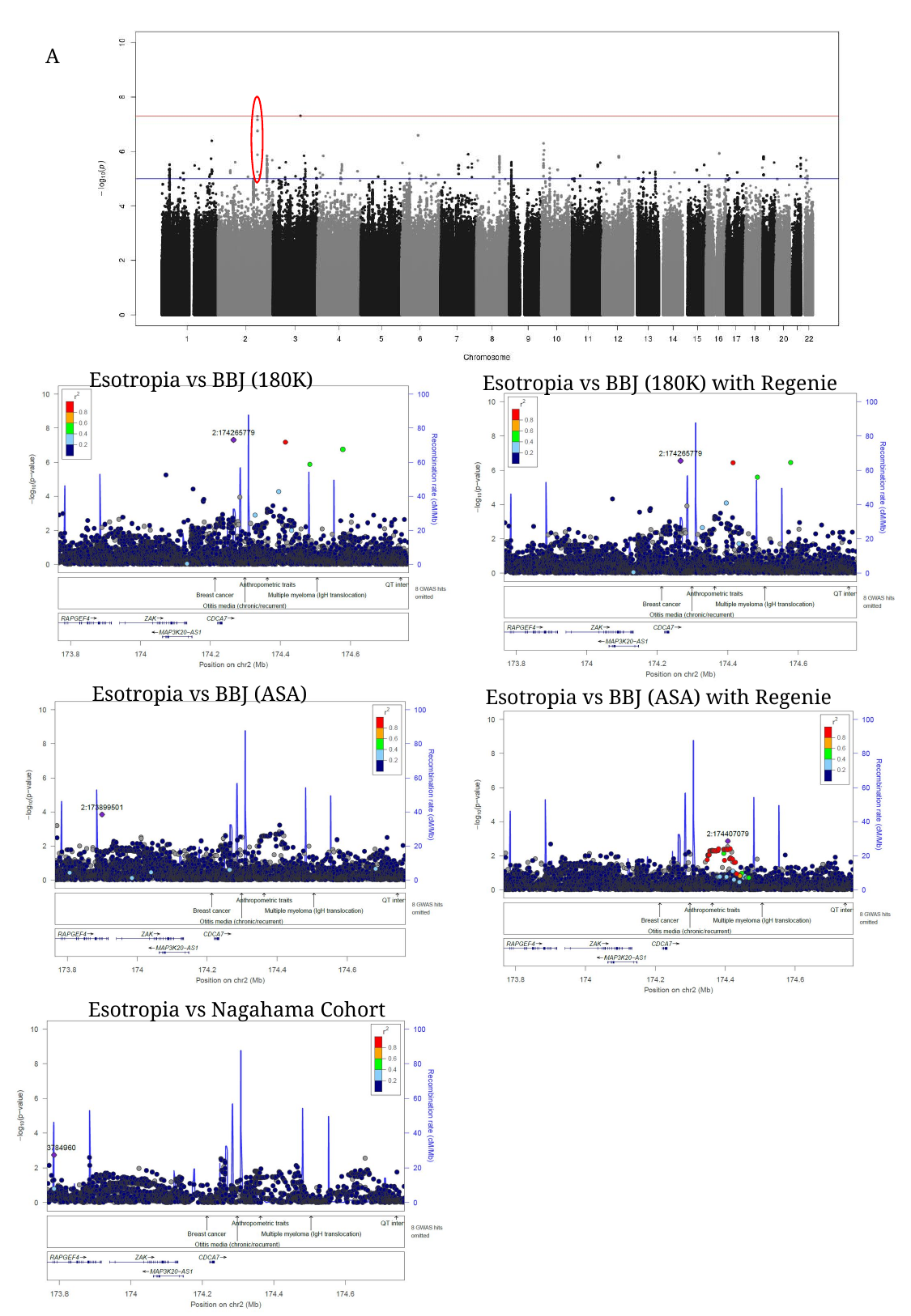

A
Esotropia vs BBJ (180K)
Esotropia vs BBJ (180K) with Regenie
Esotropia vs BBJ (ASA)
Esotropia vs BBJ (ASA) with Regenie
Esotropia vs Nagahama Cohort

## Slide 3
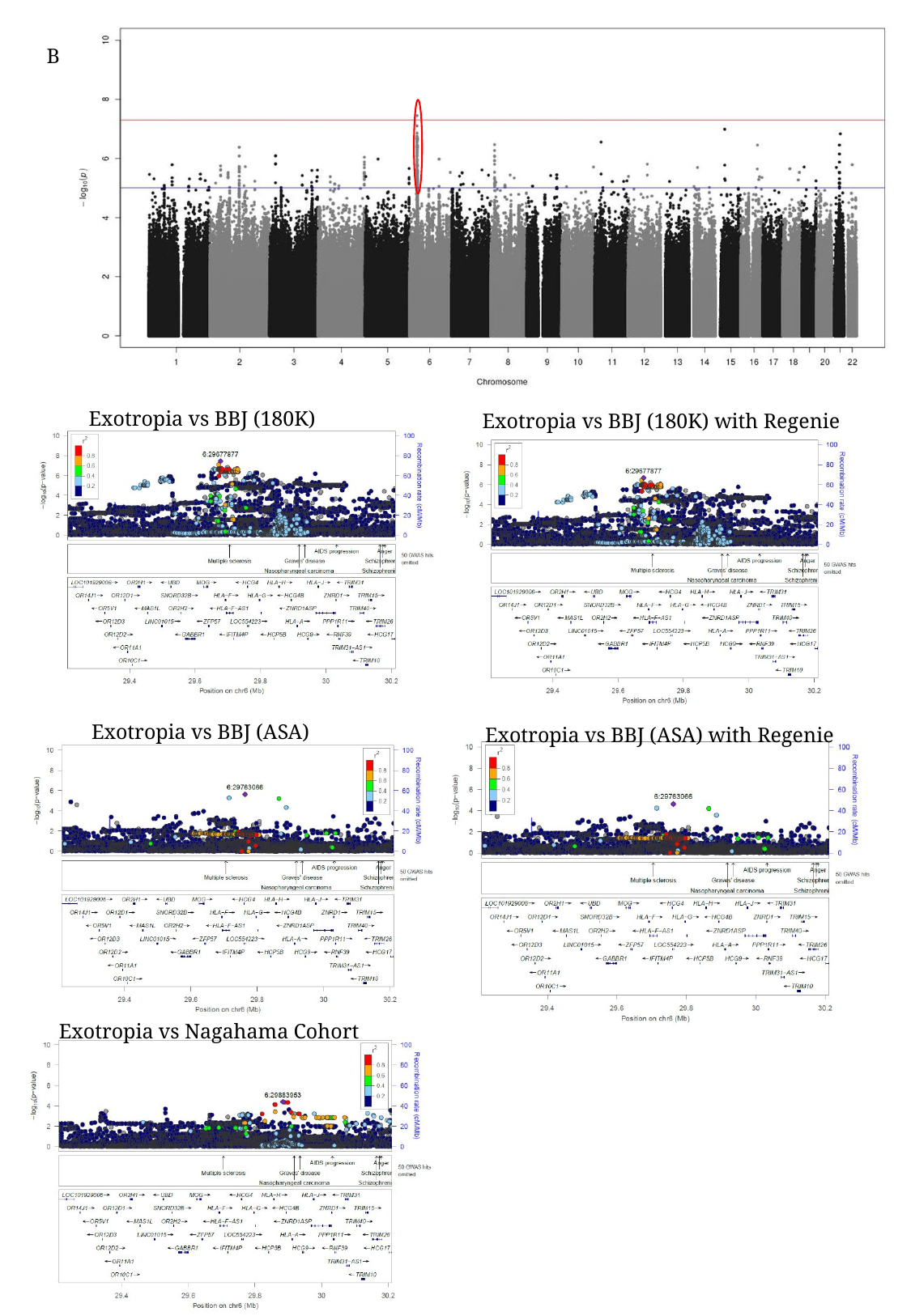

B
Exotropia vs BBJ (180K)
Exotropia vs BBJ (180K) with Regenie
Exotropia vs BBJ (ASA)
Exotropia vs BBJ (ASA) with Regenie
Exotropia vs Nagahama Cohort

## Slide 4
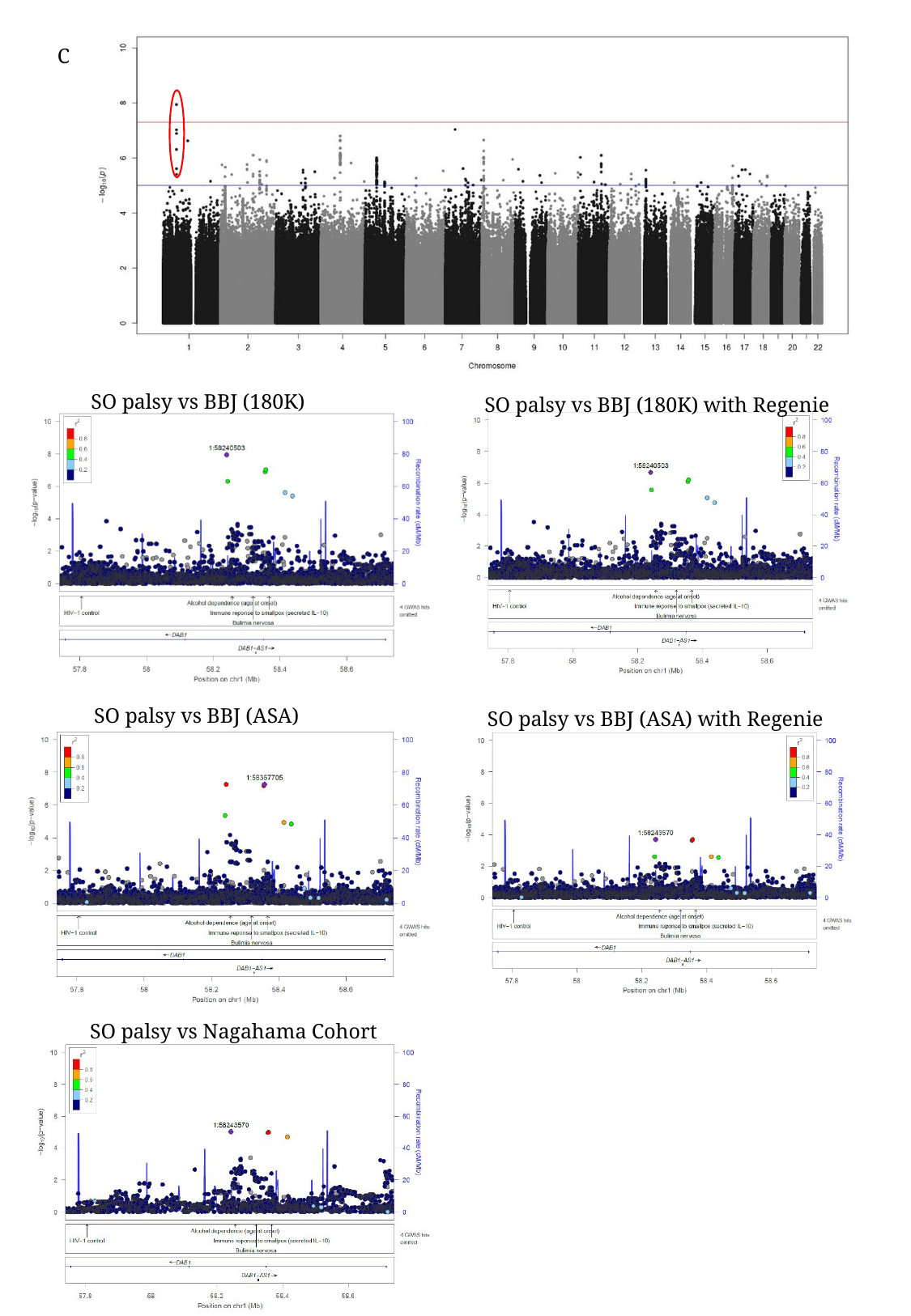

C
SO palsy vs BBJ (180K)
SO palsy vs BBJ (180K) with Regenie
SO palsy vs BBJ (ASA)
SO palsy vs BBJ (ASA) with Regenie
SO palsy vs Nagahama Cohort

## Slide 5
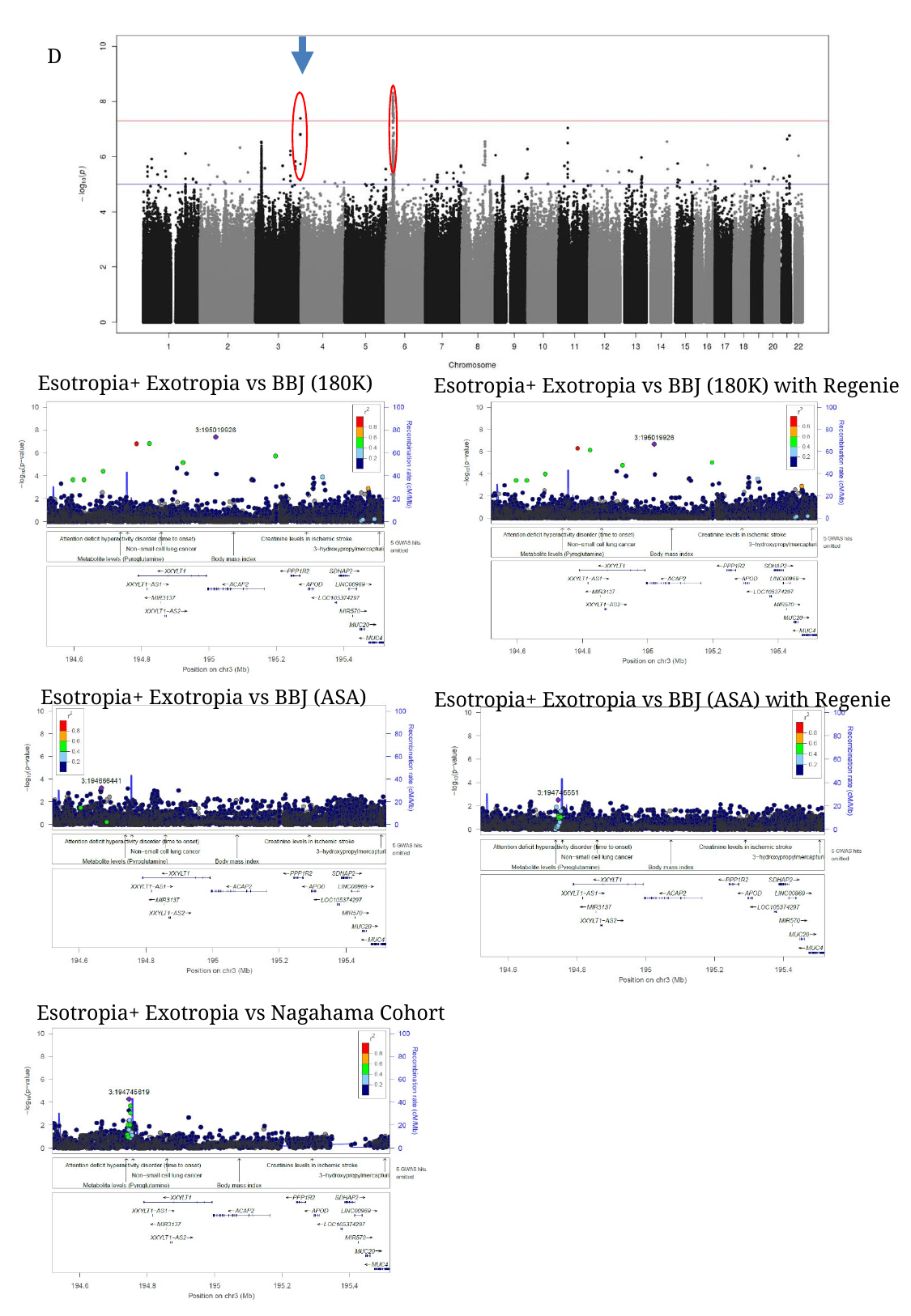

D
Esotropia+ Exotropia vs BBJ (180K)
Esotropia+ Exotropia vs BBJ (180K) with Regenie
Esotropia+ Exotropia vs BBJ (ASA)
Esotropia+ Exotropia vs BBJ (ASA) with Regenie
Esotropia+ Exotropia vs Nagahama Cohort

## Slide 6
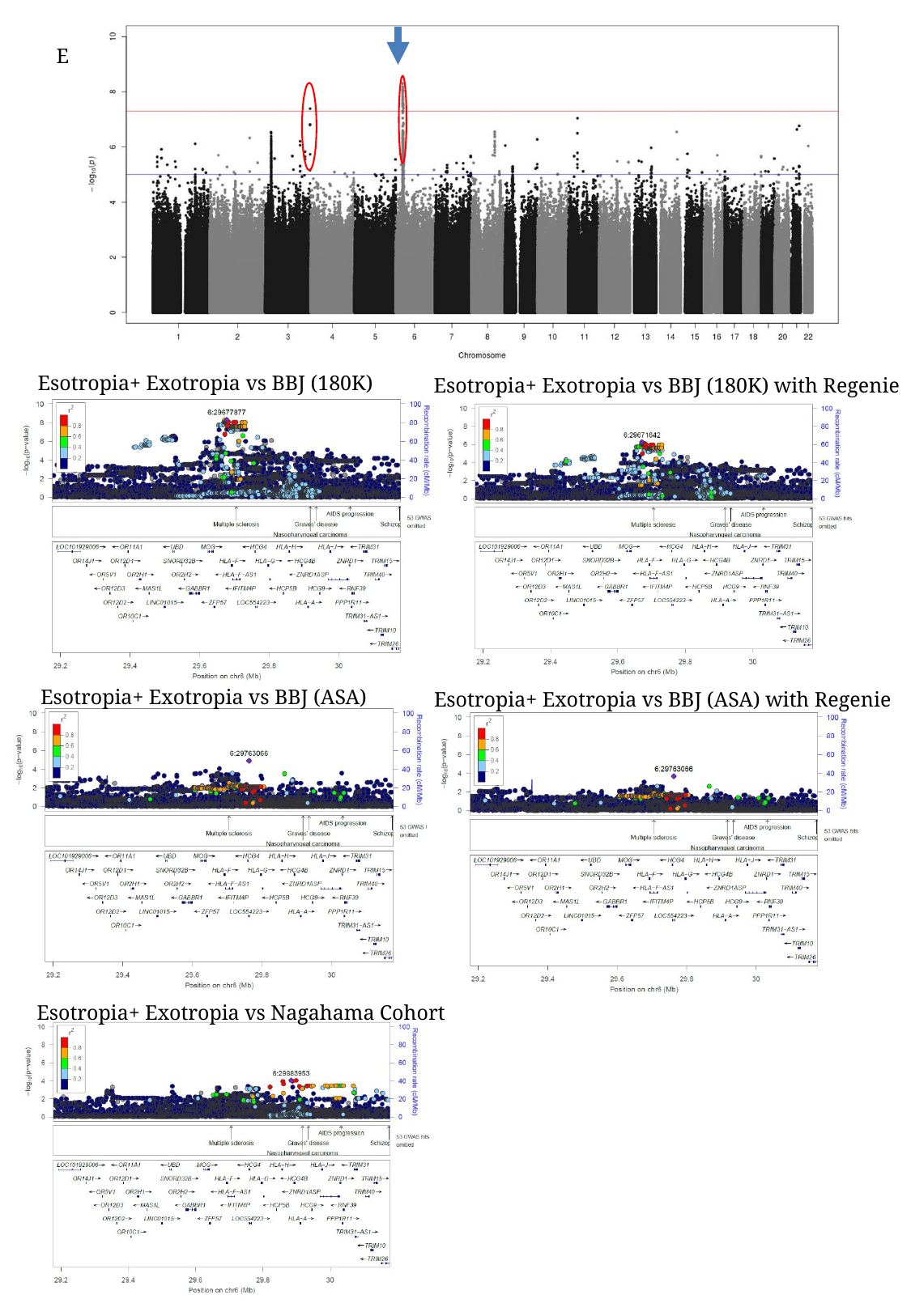

E
Esotropia+ Exotropia vs BBJ (180K)
Esotropia+ Exotropia vs BBJ (180K) with Regenie
Esotropia+ Exotropia vs BBJ (ASA)
Esotropia+ Exotropia vs BBJ (ASA) with Regenie
Esotropia+ Exotropia vs Nagahama Cohort

## Slide 7
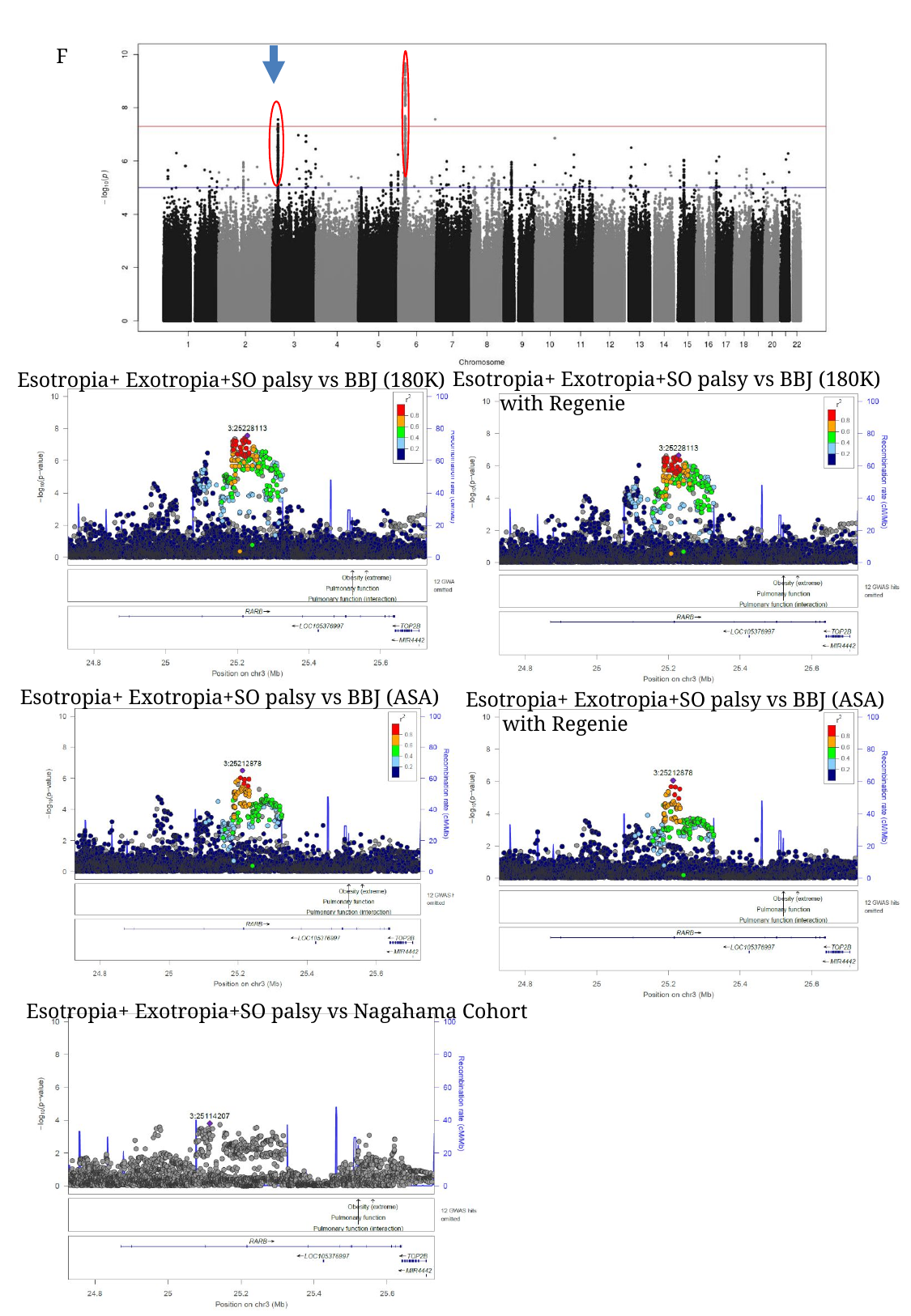

F
Esotropia+ Exotropia+SO palsy vs BBJ (180K)
 with Regenie
Esotropia+ Exotropia+SO palsy vs BBJ (180K)
Esotropia+ Exotropia+SO palsy vs BBJ (ASA)
Esotropia+ Exotropia+SO palsy vs BBJ (ASA)
 with Regenie
Esotropia+ Exotropia+SO palsy vs Nagahama Cohort

## Slide 8
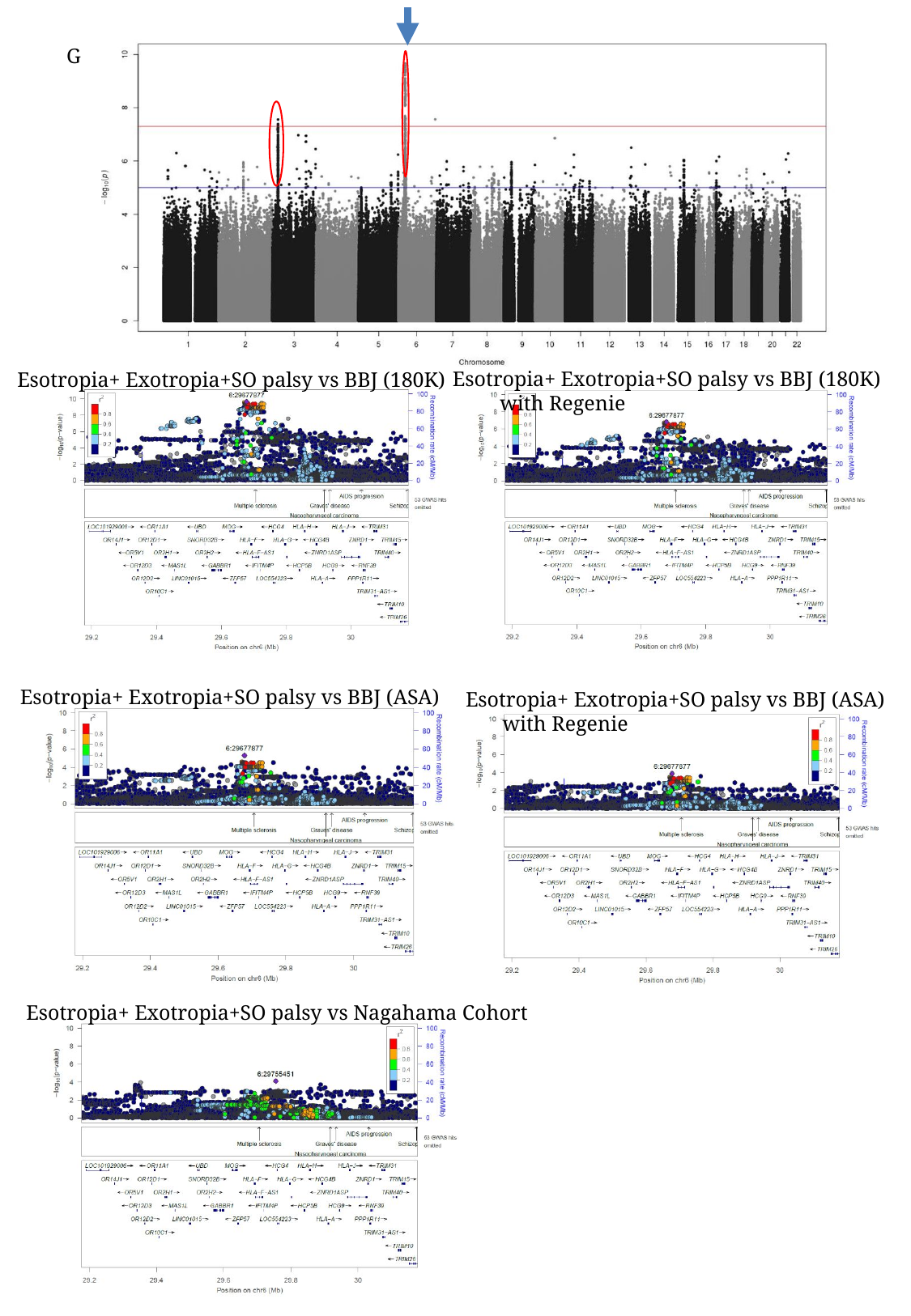

G
Esotropia+ Exotropia+SO palsy vs BBJ (180K)
 with Regenie
Esotropia+ Exotropia+SO palsy vs BBJ (180K)
Esotropia+ Exotropia+SO palsy vs BBJ (ASA)
Esotropia+ Exotropia+SO palsy vs BBJ (ASA)
 with Regenie
Esotropia+ Exotropia+SO palsy vs Nagahama Cohort
