# Supplementary material for: Genome-Wide Association Study with Three Control Cohorts of Japanese Patients with Esotropia and Exotropia of Comitant Strabismus and Idiopathic Superior Oblique Muscle Palsy
Source: Int J Mol Sci. 2024 Jun 26;25(13):6986. doi: 10.3390/ijms25136986 (PMC11241339; doi:10.3390/ijms25136986)
Supplement: Supplementary file 1 [file ijms-25-06986-s001.zip › Supplementary Figure S2 with legend.pptx]

## Slide 1
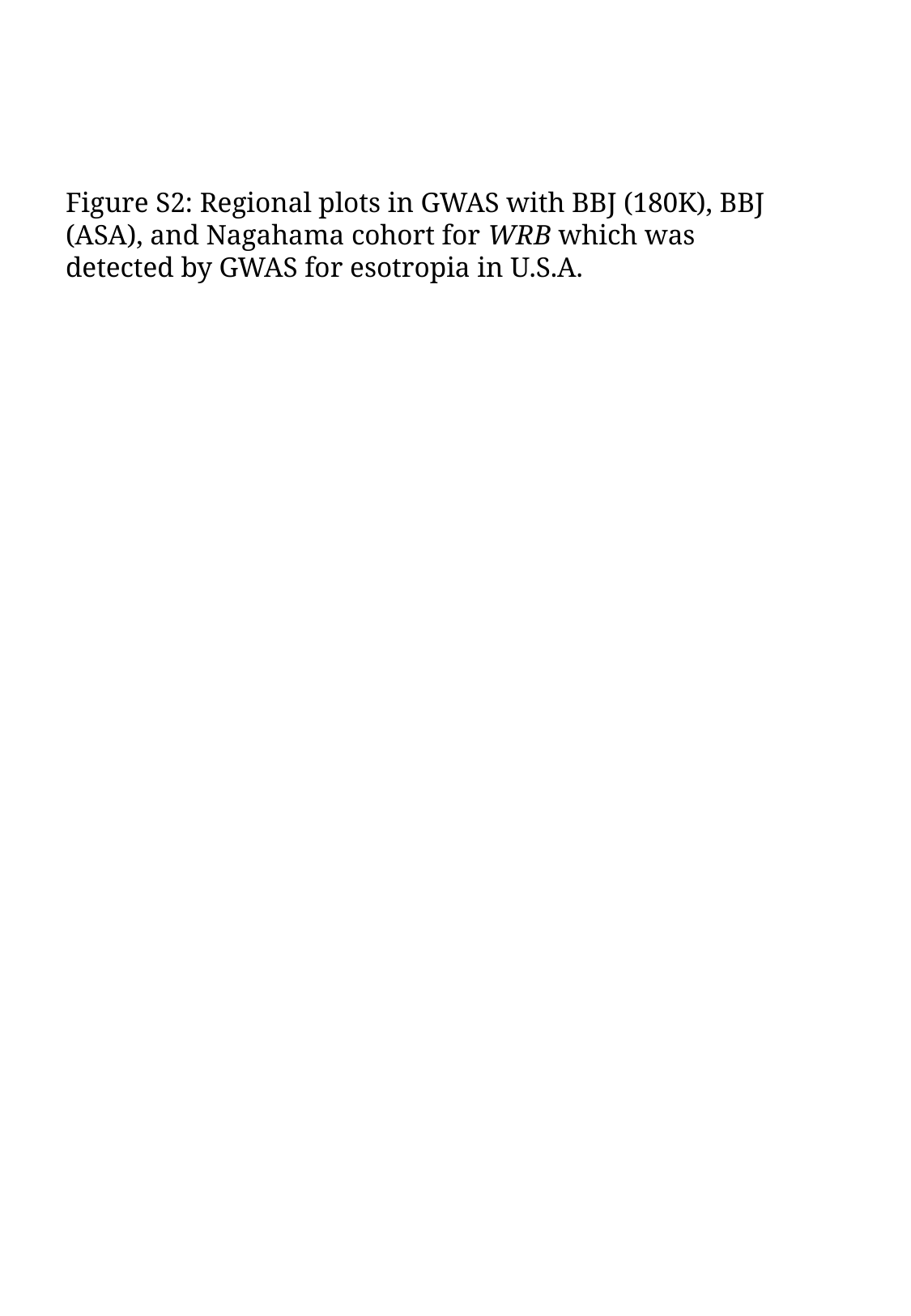

Figure S2: Regional plots in GWAS with BBJ (180K), BBJ (ASA), and Nagahama cohort for WRB which was detected by GWAS for esotropia in U.S.A.

## Slide 2
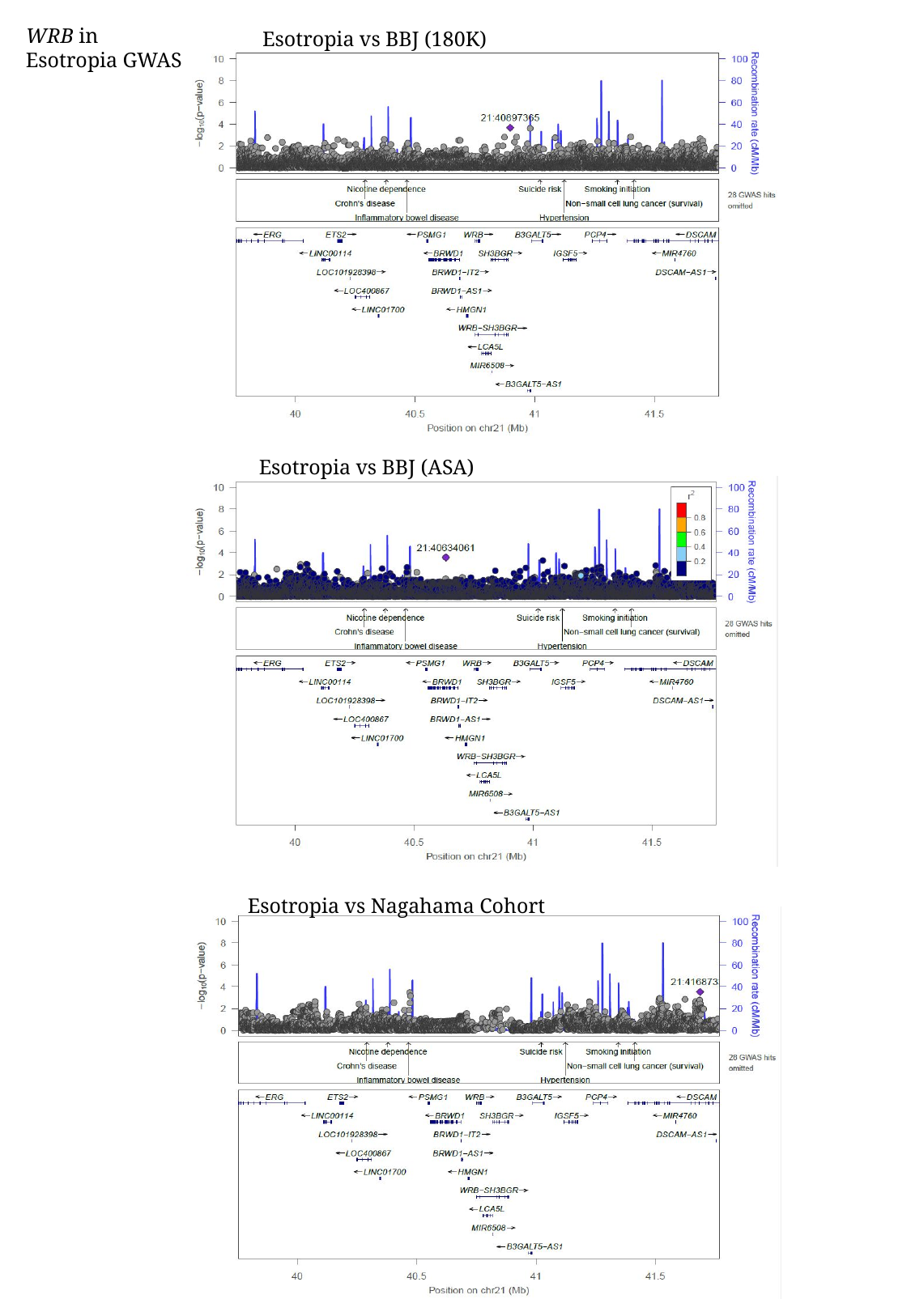

WRB in
Esotropia GWAS
Esotropia vs BBJ (180K)
Esotropia vs BBJ (ASA)
Esotropia vs Nagahama Cohort
